# Crystal structure and functional characterization of an oligosaccharide dehydrogenase from *Pycnoporus cinnabarinus* provides insights into fungal breakdown of lignocellulose

**DOI:** 10.1186/s13068-021-02003-y

**Published:** 2021-07-22

**Authors:** Gabriele Cerutti, Elena Gugole, Linda Celeste Montemiglio, Annick Turbé-Doan, Dehbia Chena, David Navarro, Anne Lomascolo, François Piumi, Cécile Exertier, Ida Freda, Beatrice Vallone, Eric Record, Carmelinda Savino, Giuliano Sciara

**Affiliations:** 1 Dipartimento di Scienze Biochimiche “A. Rossi Fanelli”, Sapienza University of Rome, Rome, Italy; 2 Istituto Pasteur-Fondazione Cenci Bolognetti, Sapienza University of Rome, Rome, Italy; 3 Consiglio Nazionale delle Ricerche (CNR) Institute of Molecular Biology and Pathology, P.le A. Moro 5, 00185 Rome, Italy; 4 Anses, INRAE, Ecole Nationale Vétérinaire d’Alfort, Université Paris-Est, UMR1161 Virologie, Maisons-Alfort, France; 5 INRAE, Aix Marseille Université, BBF UMR1163 Biodiversité et Biotechnologie Fongiques, 163 Avenue de Luminy, 13009 Marseille, France; 6 Present Address: Zuckerman Mind Brain Behavior Institute, Columbia University, New York, NY 10029 USA

**Keywords:** Oligosaccharide dehydrogenase, Redox enzymes, *Pycnoporus cinnabarinus*, X-ray crystallography, Lignocellulose degradation, Laminaribiose

## Abstract

**Background:**

Fungal glucose dehydrogenases (GDHs) are FAD-dependent enzymes belonging to the glucose-methanol-choline oxidoreductase superfamily. These enzymes are classified in the “Auxiliary Activity” family 3 (AA3) of the Carbohydrate-Active enZymes database, and more specifically in subfamily AA3_2, that also includes the closely related flavoenzymes aryl-alcohol oxidase and glucose 1-oxidase. Based on sequence similarity to known fungal GDHs, an AA3_2 enzyme active on glucose was identified in the genome of *Pycnoporus cinnabarinus*, a model Basidiomycete able to completely degrade lignin.

**Results:**

In our work, substrate screening and functional characterization showed an unexpected preferential activity of this enzyme toward oligosaccharides containing a β(1→3) glycosidic bond, with the highest efficiency observed for the disaccharide laminaribiose. Despite its sequence similarity to GDHs, we defined a novel enzymatic activity, namely oligosaccharide dehydrogenase (ODH), for this enzyme. The crystallographic structures of ODH in the sugar-free form and in complex with glucose and laminaribiose unveiled a peculiar saccharide recognition mechanism which is not shared with previously characterized AA3 oxidoreductases and accounts for ODH preferential activity toward oligosaccharides. The sugar molecules in the active site of ODH are mainly stabilized through CH-π interactions with aromatic residues rather than through hydrogen bonds with highly conserved residues, as observed instead for the fungal glucose dehydrogenases and oxidases characterized to date. Finally, three sugar-binding sites were identified on ODH external surface, which were not previously observed and might be of importance in the physiological scenario.

**Conclusions:**

Structure–function analysis of ODH is consistent with its role as an auxiliary enzyme in lignocellulose degradation and unveils yet another enzymatic function within the AA3 family of the Carbohydrate-Active enZymes database. Our findings allow deciphering the molecular determinants of substrate binding and provide insight into the physiological role of ODH, opening new perspectives to exploit biodiversity for lignocellulose transformation into fuels and chemicals.

**Supplementary Information:**

The online version contains supplementary material available at 10.1186/s13068-021-02003-y.

## Background

The woody material of plants is a complex mixture of carbon-based polymers, mainly cellulose, hemicellulose and lignin, collectively called lignocellulosic biomass or lignocellulose. Cellulose is the most abundant biopolymer on earth and lignin, a heterogenous polymer assembled from differently methoxylated aromatic alcohols, accounts for about 25% of removable organic matter in the biosphere [1]. White-rot fungi are saprotrophic organisms able to effectively and selectively degrade lignocellulose. This is achieved through a wide arsenal of enzymes secreted by the fungus, which act in synergy to perform lignin enzymatic combustion [2]. Among them, some redox enzymes are classified within the “Auxiliary Activities” (AA) group [3] of the Carbohydrate-Active enZymes (CAZy) database, a curated collection of enzymes involved in carbohydrate transformations and lignocellulolysis [4]. *Pycnoporus cinnabarinus* (syn. *Trametes cinnabarina*) is a white-rot fungus known for its very efficient lignocellulose-degrading properties, whose genome encodes for a large enzymatic arsenal of CAZymes, including lignin degrading enzymes: 5 laccases (CAZy family AA1), 9 class-II peroxidases (AA2) and 24 flavoenzymes (AA3). Among the latter, 19 belong to the glucose/aryl-alcohol oxidase/dehydrogenase group (subfamily AA3_2) [5]. This enzymatic versatility allowed *P. cinnabarinus* to stand out as a microorganism of choice for the biotransformation of aromatic compounds deriving from raw plant materials, with the aim of producing high-value products such as pharmaceuticals, antioxidants and aromas [6–9]. The use of lignin as a natural source of chemicals and biofuels represents an extremely promising target in the context of green chemistry and biorefinery, since it is currently regarded as one of the causes of lignocellulose recalcitrance to industrial treatments and as a low-grade by-product of industrial activities that employ cellulose and hemicellulose [10]. In this context, a detailed characterization of the biochemical machinery underlying lignocellulose and lignin degradation by white-rot fungi, like *P. cinnabarinus*, is required to develop novel biotechnologies for lignin valorization.

The first step of fungal lignin degradation in vivo is laccase-mediated oxidative attack, responsible for the formation of unstable radical species known as phenoxy radicals [11]; while in vitro these laccase-generated radicals lead to lignin repolymerization, some unknown physiological mechanism enables fungi to completely degrade lignin. AA3 enzymes have been proposed to play a role in reducing and therefore deactivating phenoxy radicals, by oxidizing lignin and polysaccharide degradation products [12–14].

The members of the AA3 CAZy family are FAD-dependent enzymes that belong to the glucose-methanol-choline (GMC) oxidoreductase superfamily [15, 16]. Four AA3 subfamilies have been described that account for different FAD binding modes, enzymatic mechanisms and substrate preferences [17]. Subfamily AA3_2 includes eight phylogenetically distant clades of genes, coding for enzymes of unknown function (six clades), for aryl-alcohol oxidases (EC 1.1.3.7) and dehydrogenases (1 clade), as well as for glucose oxidases (GOXs; EC 1.1.3.4) and glucose dehydrogenases (GDHs; EC 1.1.5.9) [13]. Within the latter GOX/GDH clade, finally, phylogenetic analysis suggests the existence of one group of well characterized GOXs, and three groups of GDHs [16, 17]: GDH class-I (Ascomycota), including most of the already characterized GDHs; GDH class-II (Ascomycota), phylogenetically related to class-I and including enzymes not yet characterized; and GDH class-III, including mostly proteins from Basidiomycota, but also two phylogenetically related groups of proteins from each phylum, respectively, also reminiscent in sequence to GOXs [16]. In this work we study the only enzyme characterized within the GDH class-III subclade, that we previously shown to be active on D-glucose (GLC) [14].

**Fig. 1:**
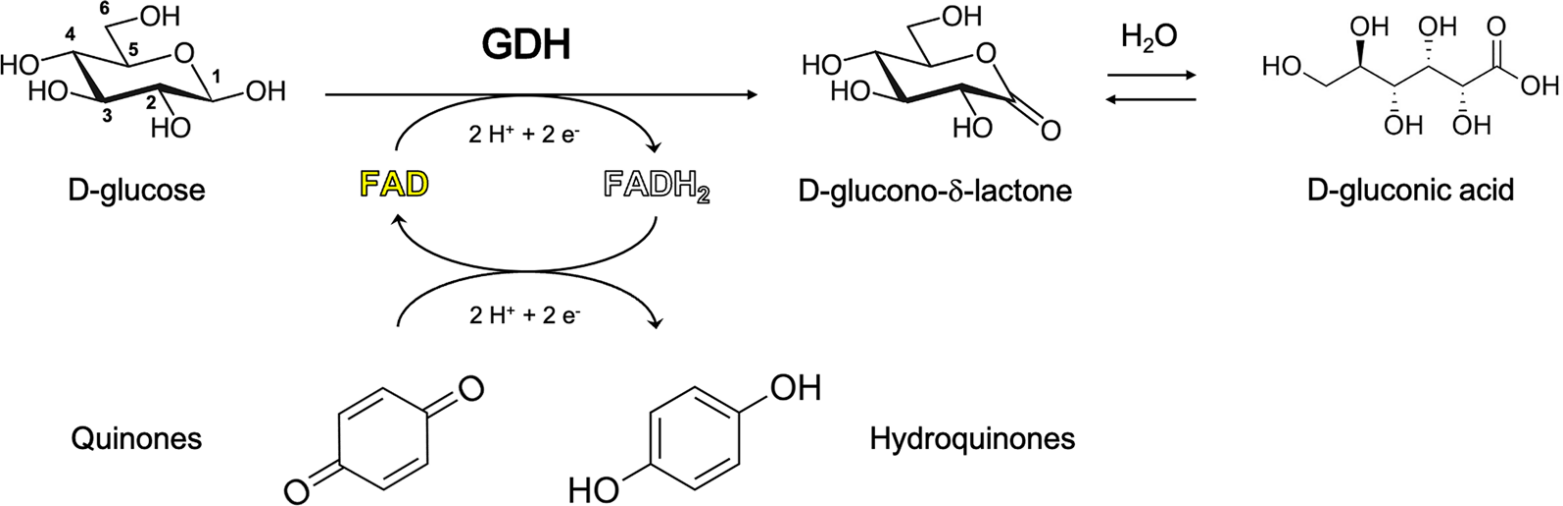
Catalytic cycle of GDHs. The enzyme in the oxidized resting state accepts electrons from a reducing sugar, like D-glucose, which is oxidized at the C1 position and converted into D-glucono-δ-lactone; this causes the protein solution to turn from yellow to colorless, due to the reduction of the FAD cofactor. In the second half-reaction electrons are transferred from reduced FADH_2_ to aromatic electron acceptors, such as quinones. The enzymatic product D-glucono-δ-lactone undergoes spontaneous hydrolysis in water

The catalytic cycle of AA3_2 enzymes is thought to consist of a hydride and proton transfer from an oxidizable substrate to a final electron and proton acceptor [18–22], and it can be divided into two half-reactions (Fig. 1). In the case of GDH and GOX, during the first half-reaction, deprotonation of GLC O1 hydroxyl triggers the transfer of two electrons and two protons to the oxidized FAD cofactor in its resting state. Once reduced, FADH_2_ is able to reverse the transfer to either oxygen (GOXs) or to a variety of aromatic electron acceptors like quinones (GDHs) [23], as shown in Fig. 1, and possibly to phenoxy radicals as mentioned above. Within the physiological scenario, the latter reaction has been proposed to inhibit lignin repolymerization in vivo [12, 13], however other biological roles have been proposed for AA3 dehydrogenases, such as providing reduced hydroquinones for lytic polysaccharide monooxygenase (LPMO) catalysis. Finally, oxidases would generate hydrogen peroxide for lytic polysaccharide monooxygenases and peroxidases [17].

In this study, we report the structural and functional characterization of a AA3_2 flavoenzyme from the white-rot fungus *P. cinnabarinus* and we give evidence for a novel enzymatic activity within the GOX/GDH clade of fungal oxidoreductases. This flavoenzyme (GenBank: CDO69819.1; UniProtKB/TrEMBL: A0A060SC37) was previously assigned as a GDH class-III for its measurable activity on GLC [14], but here we show that it acts more efficiently as an oligosaccharide dehydrogenase (ODH). We used spectrophotometric methods to evaluate substrate specificity and kinetic parameters toward a selected set of sugar substrates, observing that GLC is not the preferred substrate. ODH showed instead a marked preference for oligosaccharides in which the reducing glucosyl unit is linked to the adjacent glucose by a β(1→3) glycosidic bond, as in the disaccharide laminaribiose (G3G), which we identified as the best substrate within the selected set. We determined the crystallographic structure of ODH in the ligand-free form and in complex with GLC and G3G. Structural comparison between native and sugar-bound ODH reveals a substrate-binding mechanism which is not shared with any GDH and GOX characterized so far and which accounts for its preferred activity toward β(1→3)-containing oligosaccharides. Finally, structure–function analysis of ODH raises new questions about phylogeny and functions of fungal AA3 enzymes, providing insight into fungal lignocellulose degradation and contributing valuable information for future developments of lignin biorefinery.

## Results

### Evaluation of substrate specificity and kinetic analysis of ODH

**Fig. 2:**
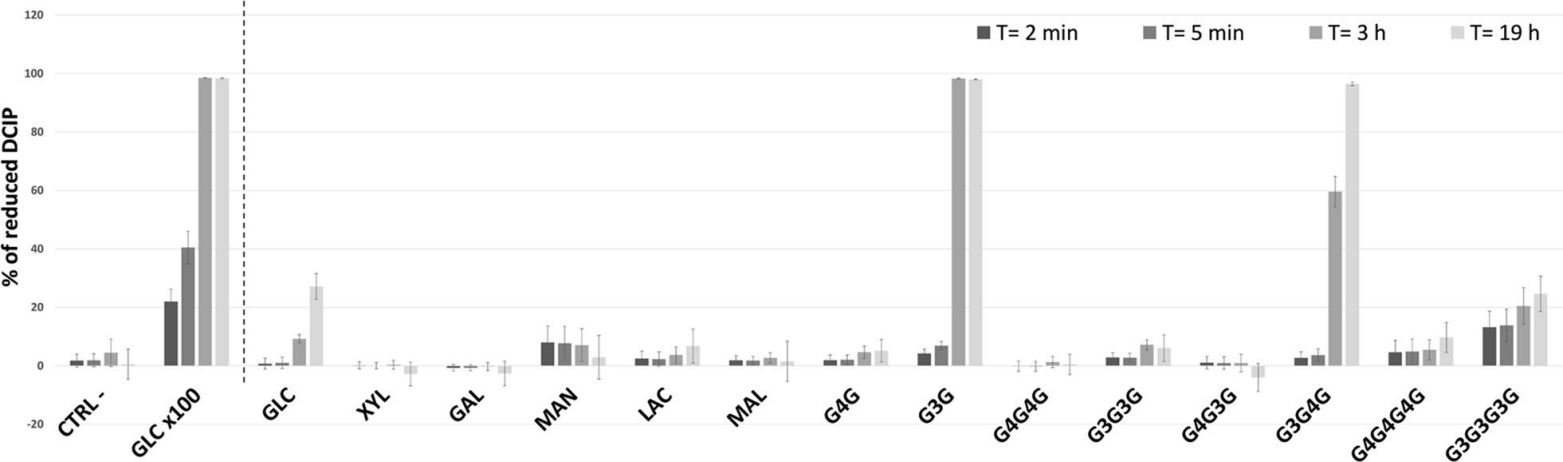
ODH substrate screening. Histograms correspond to the amount of DCIP which is reduced over time using a set of 14 sugars as electron donors for ODH reaction. Columns are colored using a grey scale code from black to light grey (*t* = 2 min to *t* = 19 h, respectively). Sugar substrates were all tested at the same concentration (2.5 mM). They include the monosaccharides D-glucose (GLC), D-xylose (XYL), D-galactose (GAL) and D-mannose (MAN), the disaccharides D-lactose (LAC), D-maltose (MAL), cellobiose (G4G) and laminaribiose (G3G), the trisaccharides cellotriose (G4G4G), laminaritriose (G3G3G), 1,3;1,4 β-glucotriose A (G4G3G) and 1,3;1,4 β-glucotriose B (G3G4G), as well as cellotetraose (G4G4G4G) and laminaritetraose (G3G3G3G). Negative (CTRL -) and positive (GLCx100) control reactions were conducted with no substrate and with 250 mM GLC, respectively, and are reported on the left of the dashed line. Absorbance decrease at 520 nm was converted into percentage of reduced DCIP, using as 100% the absorbance of a solution containing no DCIP. Error bars represent the standard deviation of three independent experiments

To gain insight into the biological function of ODH, a set of 14 sugars, differing in their carbon atom number and stereochemistry, oligomeric state and glycosidic bonds (see Additional file 1: Fig. S1), were tested by following DCIP reduction due to ODH enzymatic activity (Fig. 2). Among the inquired monosaccharides, ODH displays the highest activity toward GLC, as previously reported [14].

The screening of disaccharides revealed that ODH discriminates between different types of glycosidic linkage. More in detail, only negligible or no activity was observed if the reducing glucosyl unit was linked to the adjacent monosaccharide by a β(1→4) glycosidic bond, as in lactose (LAC) and cellobiose (G4G), or by a α(1→4) linkage, as in maltose (MAL). Conversely, pronounced enzymatic activity was detected for the β(1→3) disaccharide G3G, which behaved as the best substrate and lead to complete reduction of DCIP after 3 h, only comparable to what observed using 100 times higher GLC concentration. To evaluate ODH activity towards all glucobiose isomers with a reducing end, substrate screening was further extended to sophorose, gentiobiose, nigerose, kojibiose and isomaltose. No enzyme activity was detected, apart from gentiobiose that was oxidized at comparable rates than those observed for GLC (see Additional file 1: Fig. S2).

The analysis of trisaccharide oxidation provides further insight into substrate specificity, confirming that ODH has a preference toward sugars containing β(1→3)-linked reducing glucose, with no activity observed toward cellotriose (G4G4G) and 1,3;1,4 β-glucotriose A (G4G3G), containing a β(1→4)-bound reducing sugar unit. Moreover, ODH is active only if the second glycosidic bond starting from the trisaccharide reducing end is β(1→4) and not β(1→3), as in 1,3;1,4 β-glucotriose B (G3G4G) and laminaritriose (G3G3G), respectively. As a further confirmation of these preferences, we detected no or only negligible activity testing tetrasaccharides containing all β(1→3) and all β(1→4) linkages, namely laminaritetraose (G3G3G3G) and cellotetraose (G4G4G4G). In order to confirm the chemical nature of the enzymatic reaction products, LC–MS experiments were carried out, showing that GLC was converted by ODH to gluconolactone (not shown), as previously reported [14], and G3G to laminaribionolactone, by oxidation of glucose C1 hydroxyl (see Additional file 1: Fig. S3).

**Fig. 3:**
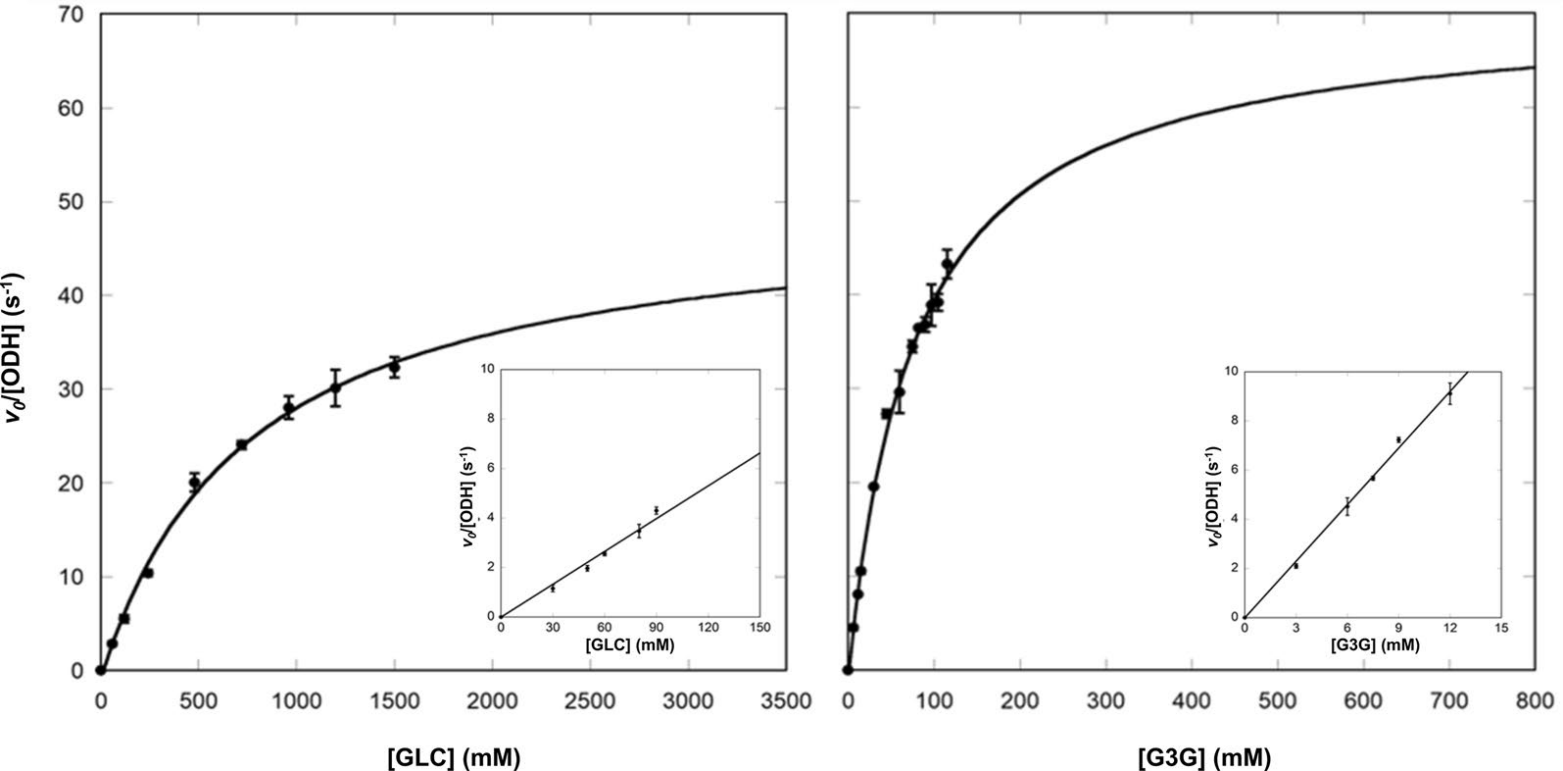
Initial rates of ODH reaction as a function of substrate concentration. ODH enzymatic kinetic assays were performed in the presence of GLC (*left panel*) and G3G (*right panel*) at different concentrations. Data were fitted to the standard Michaelis–Menten hyperbolic equation. Insets represent a linear plot of initial velocities of ODH at low substrate concentrations in the linear region of the Michaelis–Menten hyperbola. Error bars represent the standard deviation of three independent experiments

More extensive enzymatic characterization was carried out using G3G, the best substrate, in comparison to GLC. Unfortunately for both substrates, it was impossible to measure the maximal rate of the reaction. Indeed, even though the enzymatic kinetics performed with GLC seems to follow a simple Michaelis–Menten model up to 1.5 M GLC (Fig. 3), at higher concentrations it displays a deviation from the hyperbola equation, as the measured initial rate decreased with increasing GLC concentrations (see Additional file 1: Fig. S4). Regarding G3G, completion of the kinetic curve was prevented by substrate solubility (150 mM), and we did not achieve enzyme saturation with the highest measurable concentration of G3G (115.5 mM). Therefore, apparent steady-state kinetic parameters (Table 1) were obtained from fitting the standard Michaelis–Menten equation up to 1.5 M GLC and 115.5 mM G3G (Fig. 3).

**Table 1:** Apparent ODH kinetic constants for GLC and G3G

	*K*_M_ (mM)	*k*_cat_ (s^−1^)	*k*_cat_/*K*_M_* (M^−1^ s^−1^)	*k*_cat_/*K*_M_** (M^−1^ s^−1^)
GLC	755 ± 110	50 ± 3	67 ± 10	47 ± 1
G3G	77 ± 10	71 ± 4	917 ± 129	777 ± 21

^*^k_cat_/*K*_M_ calculated from the apparent *K*_M_ and *k*_cat_ estimated from fitting the Michaelis–Menten hyperbola to data points (Fig. 3)

^**^k_cat_/*K*_M_ calculated as the slope of the tangent of the Michaelis–Menten hyperbola in its linear region (insets in Fig. 3)

The apparent K_M_ estimated for GLC is comparable to what reported previously [14]. Comparative analysis of the resulting apparent kinetic parameters indicates tenfold higher K_M_ values toward GLC with respect to G3G, but comparable maximum turnover rates (*k*_cat_) (Table 1). As a result, the specificity constant (*k*_cat_/*K*_M_) derived for G3G exceeds more than 13 times the one estimated for GLC. To support this observation, we estimated the relative specificity of ODH for GLC and G3G by measuring the initial rates in the linear region of the Michaelis–Menten plot, in which the substrate concentration is significantly smaller than the measured K_M_ values. In these conditions, the slope of the tangent of the Michaelis–Menten hyperbola at its origin corresponds to *k*_cat_/*K*_M_, and it is derived experimentally (Fig. 3, insets). The specificity constants determined by both approaches are consistent (Table 1) and confirm higher ODH specificity toward G3G. The apparent values of the kinetic parameters suggest that the consumption rate of the two substrates is very similar, and that enhanced catalytic efficiency is due to preferential binding of G3G. A simple substrate inhibition model did not allow to satisfactorily fit the initial velocity data including high GLC (up to 3 M) points (see Additional file 1: Fig. S4). At these extreme GLC concentrations, changes in refractive index and viscosity may also affect the DCIP extinction coefficient [24], whereas increased viscosity may negatively affect catalytic efficiency, due to higher molecular friction in solution and decreased enzyme conformational freedom [25].

Finally, it should be noted that all saccharide solutions used in our experiments are, naturally, a mixture of the corresponding α and β anomers. For GLC, the latter is almost twice more concentrated than the former at the equilibrium. As suggested by the crystal structures of ODH in complex with substrates (next sections), the enzyme seems to discriminate between α and β anomers, and indeed initial enzymatic velocities on freshly dissolved α-GLC were about half those on pre-equilibrated GLC solutions (see Additional file 1: Fig. S5).

### Structural features of ligand-free ODH

ODH crystals belong to the P2_1_2_1_2_1_ space group and contain one protein molecule per asymmetric unit; as such, no twofold axis nor non-crystallographic symmetries are observed. This is consistent with ODH being a monomer, as previously observed for GDH from *Aspergillus flavus* (*Af*GDH) [20], whereas GOXs from Ascomycota are known to be dimeric [18, 19]. ODH crystals are bright yellow, suggesting that the FAD cofactor is in the oxidized resting state. Data collection and refinement statistics of all structures are summarized in Table 2.

**Table 2:** X-ray diffraction data collection and structure refinement statistics. Values in parentheses are for the highest-resolution shell

	Ligand-free ODH 6XUT	ODH-GLC 6XUU	ODH-G3G 6XUV
Data collection			
Space group	P2_1_2_1_2_1_		
Unit-cell dimensions (Å)	a = 48.87 b = 61.59 c = 195.09		
Resolution range (Å)	97.55–1.43 (1.57–1.43)	49.04–1.57 (1.67–1.57)	97.33–1.75 (2.02–1.75)
Number of observations	1,391,283 (295,026)	1,064,709 (190,424)	770,177 (268,996)
Unique reflections	128,942 (25,836)	103,996 (29,033)	59,350 (20,470)
Completeness (%)	99.9 (99.7)	99.6 (98.8)	99.9 (99.8)
Redundancy	12.7 (11.4)	6.6 (6.6)	13.0 (13.1)
*I*/σ(*I*)	14.5 (1.72)	12.0 (1.36)	7.0 (1.97)
*R*_merge_^a^ (%)	7.1 (139.0)	9.0 (141.0)	17.5 (156.1)
CC_1/2_	100 (79.5)	99.9 (55.8)	99.9 (80.8)
Wilson B-value (Å^2^)	22.0	28.6	25.9
Refinement			
Resolution range (Å)	97.55–1.60	49.04–1.57	97.33–1.75
Protein molecules per asymmetric unit	1	1	1
*R*_work_/*R*_free_^b^	0.165/0.196	0.162/0.189	0.168/0.207
Deviations from ideal geometry			
Bond (Å)	0.0132	0.0127	0.0138
Angles (Å)	1.713	1.856	2.005
Ramachandran plot (%) Favored/allowed/outliers	95.4/4.6/0	96.25/3.75/0	96.25/3.75/0
Mean B-factors (Å^2^)			
Protein	42.2	36.9	48.7
FAD/GLC/G3G	33.4/-/-	28.8/47.7/-	40.1/-/61.7
Water/sulfate	41.8/55.9	42.7/85.9	52.6/105.3
Number of atoms			
Protein	5023	4902	5059
FAD/GLC/G3G	53/-/-	53/108/-	53/-/115
Water/sulfate	466/40	363/20	303/15

^a^$R_{{{\text{merge}}}} = \mathop \sum \limits_{i} \mathop \sum \limits_{j} |~I_{{i,j}} - I_{j} |/\mathop \sum \limits_{i} \mathop \sum \limits_{j} I_{{i,j}}$, where *i* runs over multiple observations of the same intensity, and *j* runs over all crystallographically unique intensities.

^b^$R_{{{\text{work}}}} = \sum \left| {\left| {F_{{{\text{obs}}}} } \right| - \left| {F_{{{\text{calc}}}} } \right|} \right|~/~\sum \left| {F_{{{\text{obs}}}} } \right|/\mathop \sum \limits_{i} \mathop \sum \limits_{j} I_{{i,j}}$, where |*F*_obs_|> 0. *R*_free_ is based on 5% of the data randomly selected and is not used in the refinement.

**Fig. 4:**
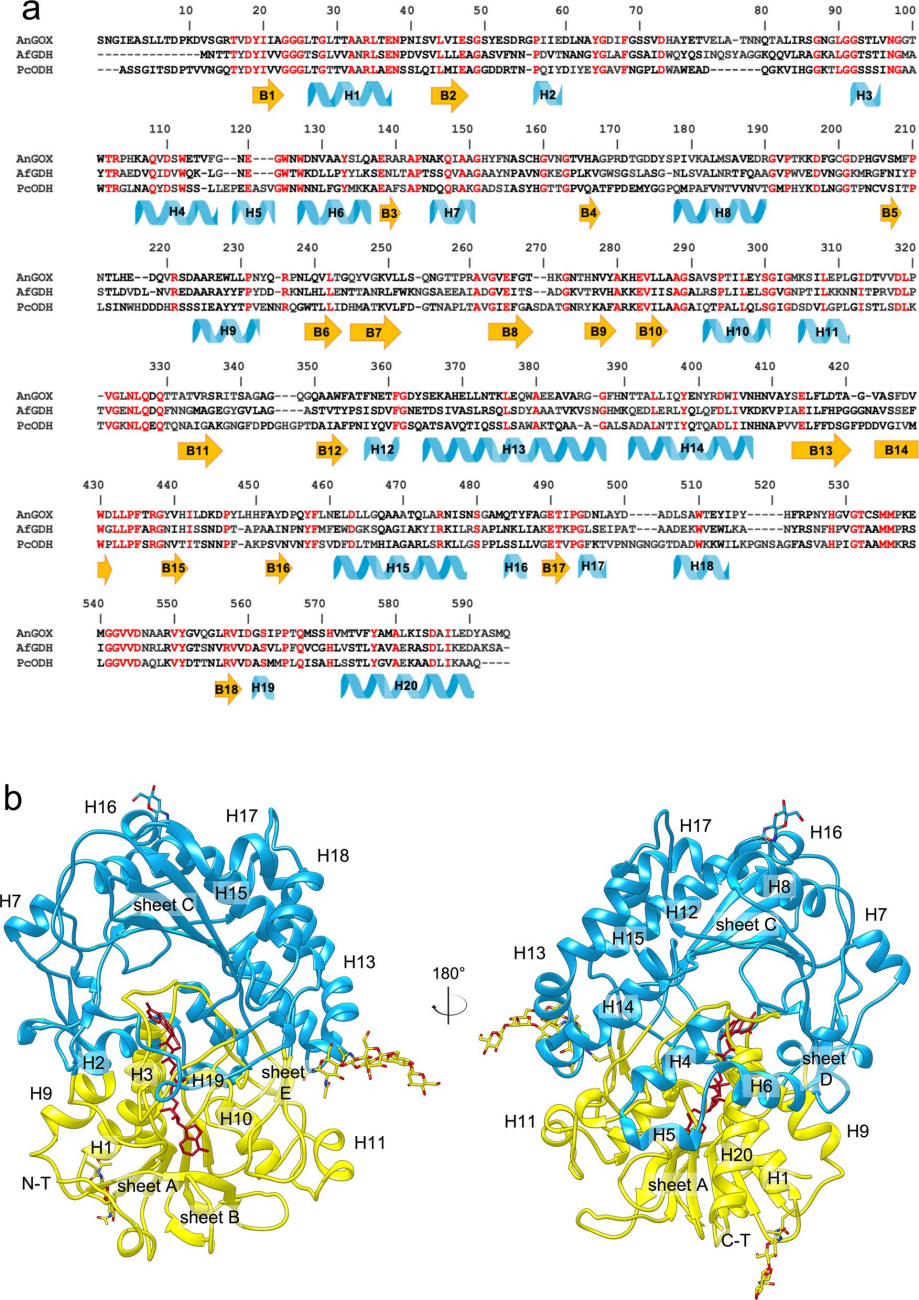
Overall structure of ODH and sequence alignment with homologous enzymes of known structure. **a** Sequence alignment of ODH, *Af*GDH and *An*GOX based on structural superposition. Numbers refer to ODH sequence; conserved residues are colored in red. Secondary structure elements are assigned for ODH: β-strands are in orange and α-helices in light blue. **b** Ribbon representation of the whole three-dimensional structure of ligand-free ODH, with secondary structures assignment for β-sheets and α-helices (H). The FAD-binding domain is shown in yellow, the substrate-binding domain in light blue, the FAD cofactor in dark red sticks. Glycosylations are represented in sticks, with C atoms in yellow or light blue, O in red and N in blue

The final structure of ligand-free ODH (PDB entry 6XUT), that was refined up to 1.6 Å resolution, is shown in Fig. 4, together with secondary structure assignment and with structural alignment to *Af*GDH (PDB: 4YNT) and GOX from *Aspergillus niger* (*An*GOX; PDB: 1CF3). Both AfGDH and AnGOX are closely related to ODH and represent the reference structures of AA3_2 enzymes from the GOX/GDH clade. Structure-based sequence alignment of ODH with the two enzymes (Fig. 4) indicates that *Af*GDH and *An*GOX share, respectively, 36.4 and 34.3% identity with ODH, as well as 1.2 Å and 1.3 Å average r.m.s.d. of Cα atom positions. Conservation of key ODH residues throughout the 7 subclades of the GOX/GDH group of AA3_2 was also analyzed (see Additional file 1: Fig. S6). The overall structure of ODH consists of 20 α-helices and 18 β-strands organized in two domains, the FAD-binding domain and the substrate-binding domain (Fig. 4), as in all GMC oxidoreductases [18–20]. The β-strands are arranged in 5 β-sheets named following the nomenclature introduced for *An*GOX [18].

The FAD-binding domain is formed by the five-stranded parallel β-sheet A (B1, B2, B6, B10, B18) that is sandwiched between the three-stranded antiparallel β-sheet B (B7, B8, B9) and three α-helices (H1, H9, H20); the βαβ -motif involved in FAD binding is formed by B1, H1 and B2.

The substrate-binding domain consists of the central six-stranded antiparallel β-sheet C (B5, B11, B12, B13, B14, B17), roofing the FAD-binding domain at the level of the FAD isoalloxazine ring and crowned by seven α-helices (H8, H12, H13, H14, H15, H17, H18). We can depict β-Sheet C as a roof which delimits a deep pocket (ODH active site) by laying on a “floor” that is formed by the FAD-binding domain and that also exposes the catalytically active moiety (isoalloxazine ring) of the FAD cofactor to the cavity. The FAD-binding domain and the substrate-binding domain are linked through two types of connections: one consists of three structured extended segments connecting the two domains, the other one involves three helices (H4, H5, H6) that protrude from the substrate-binding domain and lean on the surface of the FAD-binding one, and two extended two-stranded β-sheets (β-sheet D, parallel, and β-sheet E, antiparallel), located at the interface between domains and hooking them together.

The amino acid sequence of ODH contains three potential N-glycosylation sites, Asn38, Asn188 and Asn439, which could be predicted by the consensus sequence Asn-X-Ser/Thr and that are all visible in the crystallographic structure (Fig. 4B).

The FAD molecule is non-covalently bound to the protein and it occupies a narrow channel lined by ordered regions, mainly the loop connecting β-strand B1 and helix H1, part of a βαβ-motif. The electron density map of the FAD isoalloxazine ring clearly shows a distortion from planarity, which is expected for reduced FAD. To unequivocally point out the redox state of the cofactor, initial structure refinement trials were performed modelling either reduced (bent) or oxidized (planar) FAD; the resulting electron density maps is consistent with the presence of the cofactor in the oxidized conformation, despite the distortion from planarity. In fact, the pyrimidine moiety is ~ 11° bent toward the FAD-binding domain with respect to the pteridine plane. A bent conformation has already been observed for oxidized FAD in other GMC oxidoreductases, where the protein backbone architecture in the proximity of FAD, and in particular a conserved asparagine, restrains the cofactor geometry, causing a distortion from planarity of the isoalloxazine ring [18–20, 26]. This interpretation is also valid for ODH, where Asn97 (see Additional file 1: Fig. S7), a conserved residue within AA3 enzymes from the GOX/GDH clade (see Additional file 1: Fig. S6), points toward the central part of the isoalloxazine ring, sticking out from the “floor” of the active site cavity (FAD-binding domain). Asn97 establishes a hydrogen bond with Ser573, partially conserved within the GOX/GDH subclade (see Additional file 1: Fig. S6), and hydrogen bonds with FAD and ODH backbone atoms. Preference for short side chain residues in the three positions Gly98, Ala99 and Ala100 is also observed within the GOX/GDH clade (see Additional file 1: Fig. S6), possibly allowing stabilization of the bent pyrimidine moiety through hydrogen bonding with backbone atoms (see Additional file 1: Fig. S7). A bent conformation of oxidized FAD is thought to allow switching from the oxidized to the reduced form with minor conformational rearrangements, thus resulting in a reduced energy difference between the two states and in the modulation of FAD redox potential in favor of the reduced state.

ODH active site is located at the bottom of a large funnel-shaped cavity and it is directly accessible to the solvent. As in other GMC oxidoreductases, we identified the catalytic pair His528/His571, positioned in the proximity of FAD, on the *re*-face of the isoalloxazine ring. The imidazole rings of His528 and His571 are oriented and stabilized by hydrogen bonds with Gln329 and Glu414, respectively, conserved within the GOX/GDH clade (see Additional file 1: Fig. S6), and with the FAD reactive N5 atom through a shared water molecule (see Additional file 1: Fig. S7). Except for these histidines and Gln331, most of the residues contributing to the active site cavity are either hydrophobic or possess aromatic side chains, such as Tyr64, Phe416 and Trp430 (see Additional file 1: Fig. S7).

### Structures of ODH bound to substrates: the substrate-binding loop

**Fig. 5:**
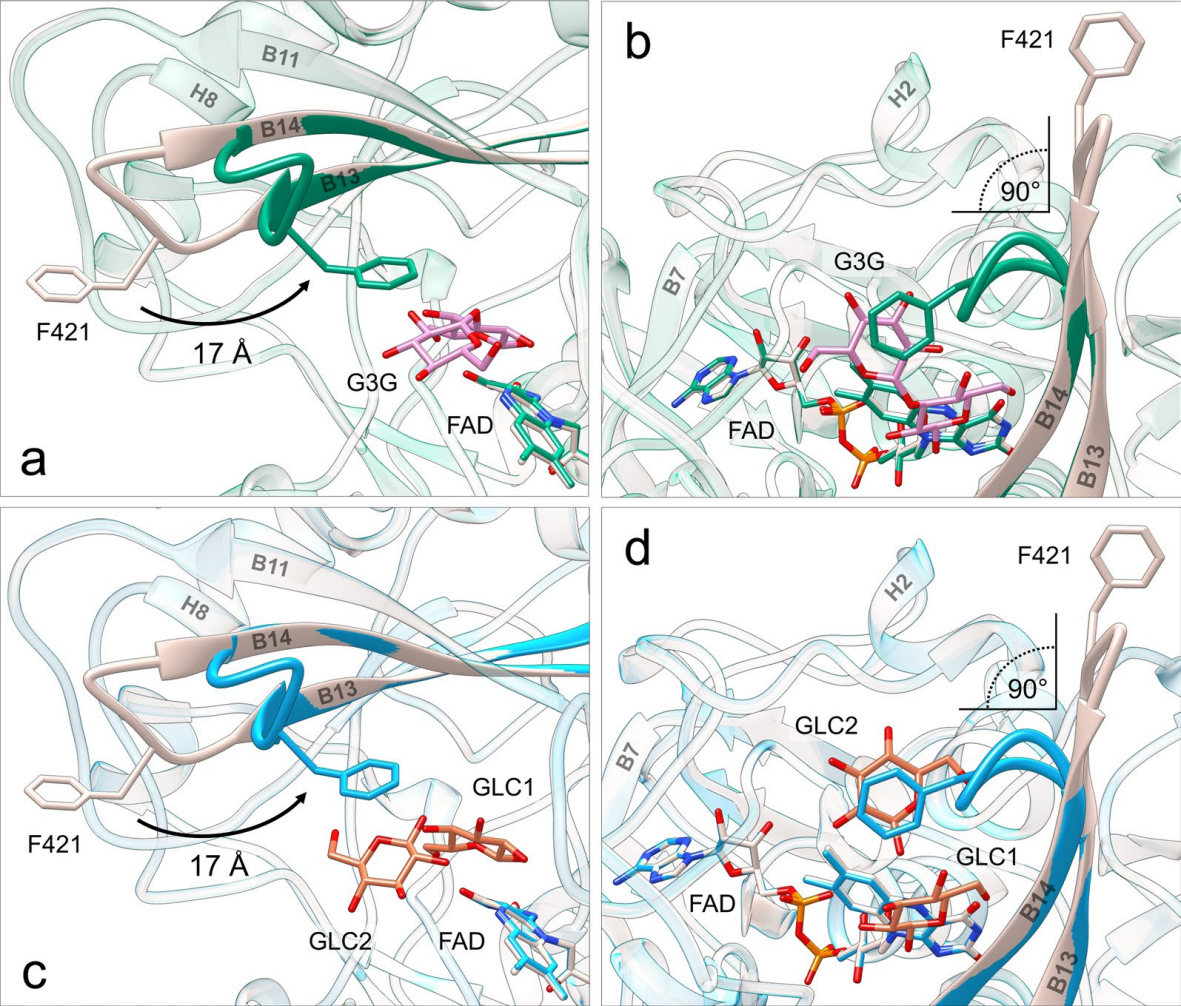
Conformational rearrangement of the substrate-binding loop upon sugar binding. Superposition of ligand-free ODH (gray) and ODH-G3G (green): **a** side view (perpendicular to the loop hinge axis), **b** top view (along the loop hinge axis). Superposition of ligand-free ODH (gray) and ODH-GLC (light blue) structures: **c** side view, **d** top view. G3G and GLC C atoms are in pink and orange, respectively, O atoms in red and N atoms in blue. For clarity sake, only β anomers are represented. Phe421 (also shown in sticks) is exposed to the bulk in the ligand-free structure and shifts ~ 17 Å toward the active site upon binding of sugars, establishing CH-π interactions with the non-reducing glucosyl unit of G3G, or with GLC2 in ODH-GLC. This movement causes the substrate-binding loop to rotate by ~ 90° and wrap toward the active site

**Fig. 6:**
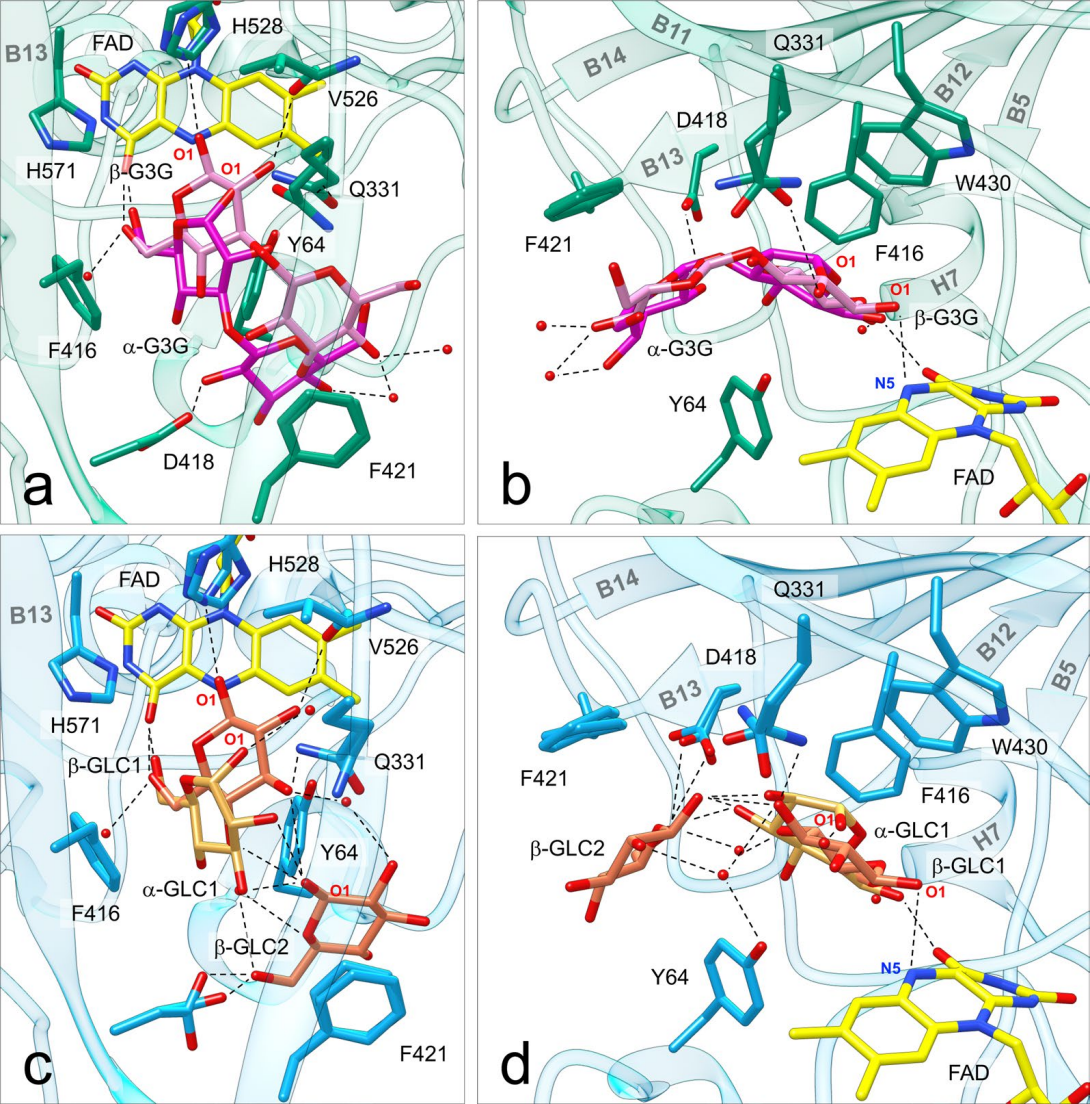
Sugar substrates bound to ODH active site. **a** Top view (perpendicular to the isoalloxazine plane) and **b** side view of ODH-G3G: the protein is shown in green, the FAD cofactor in yellow, G3G in pink (β anomer) or magenta (α anomer). **c** Top view and **d** side view of ODH-GLC: the protein is shown in light blue, the FAD cofactor in yellow, GLC in orange (β anomer) or gold (α anomer); O atoms are in red, N atoms in blue. In both structures the β anomer is closer to the FAD cofactor than the α anomer, and oriented with hydrogen bonds between the reactive O1 atom and His528 and the FAD N5 atom. Both sugars are sandwiched on top of Tyr64 by the aromatic residues Phe421, Phe416 and Trp430. Water molecules are depicted as red spheres. Hydrogen bonds (distance < 3.2 Å) are represented with dotted lines

Structures of ODH-G3G (PDB entry 6XUV) and ODH-GLC (PDB entry 6XUU) were obtained by soaking and refined up to 1.75 and 1.57 Å resolution, respectively (Table 2). Overall, ODH–sugar complexes show very few structural differences compared to the ligand-free form: pairwise superposition onto the structure of ligand-free ODH yields average Cα r.m.s.d. values of 0.41 Å and 0.34 Å, respectively. The analysis of r.m.s.d. as a function of the residue number, however, shows Cα atoms displacements exceeding 10 Å for residues forming the B13-B14 turn, to which we refer as the “substrate-binding loop” (residues 419–424, Fig. 5). While this region points toward the solvent in ligand-free ODH contributing to a wide-open active site, in sugar-bound ODH the “substrate-binding loop” restricts the active site access, bending inwards (Fig. 5). This pronounced conformational change consists of a 90° bending around two hinges (Gly420 and Asp424), that allow backbone reorientation around fixed Cα atom positions. It results into a large displacement toward the interior of the active site of three amino acids (Phe421, Pro422 and Asp423), whose tight geometry is maintained in both conformations, probably relying on the cis-proline at the position 422. Notably Phe421, exposed to the solvent in ligand-free ODH, moves ~ 17 Å towards the inside of the active site upon sugar binding (Fig. 5), clamping either G3G non-reducing glucosyl unit or a GLC molecule (GLC2) against Tyr64 (Fig. 6 and see Additional file 1: Fig. S8). For more details about residues and interactions contributing to substrate-binding loop conformations see Additional file 2: Sect. 1. Within the GOX/GDH clade, the substrate-binding loop seems to be conserved in the ODH and ODH-like subclades from Basidiomycetes, with different loop structures in the Ascomycete subclades (see Additional file 1: Fig. S6).

### Structures of ODH bound to substrates: the active site

No significant conformational changes are observed in other residues lining the active site. In the sugar-bound forms the FAD isoalloxazine ring shows a more pronounced distortion from planarity, with a bending angle of ~ 16° between the pyrimidine and the pteridine moieties (instead of ~ 11° observed in the ligand-free form) (Fig. 5), which may be an indication of the cofactor getting reduced upon crystal soaking with the oxidizable substrates.

**Fig. 7:**
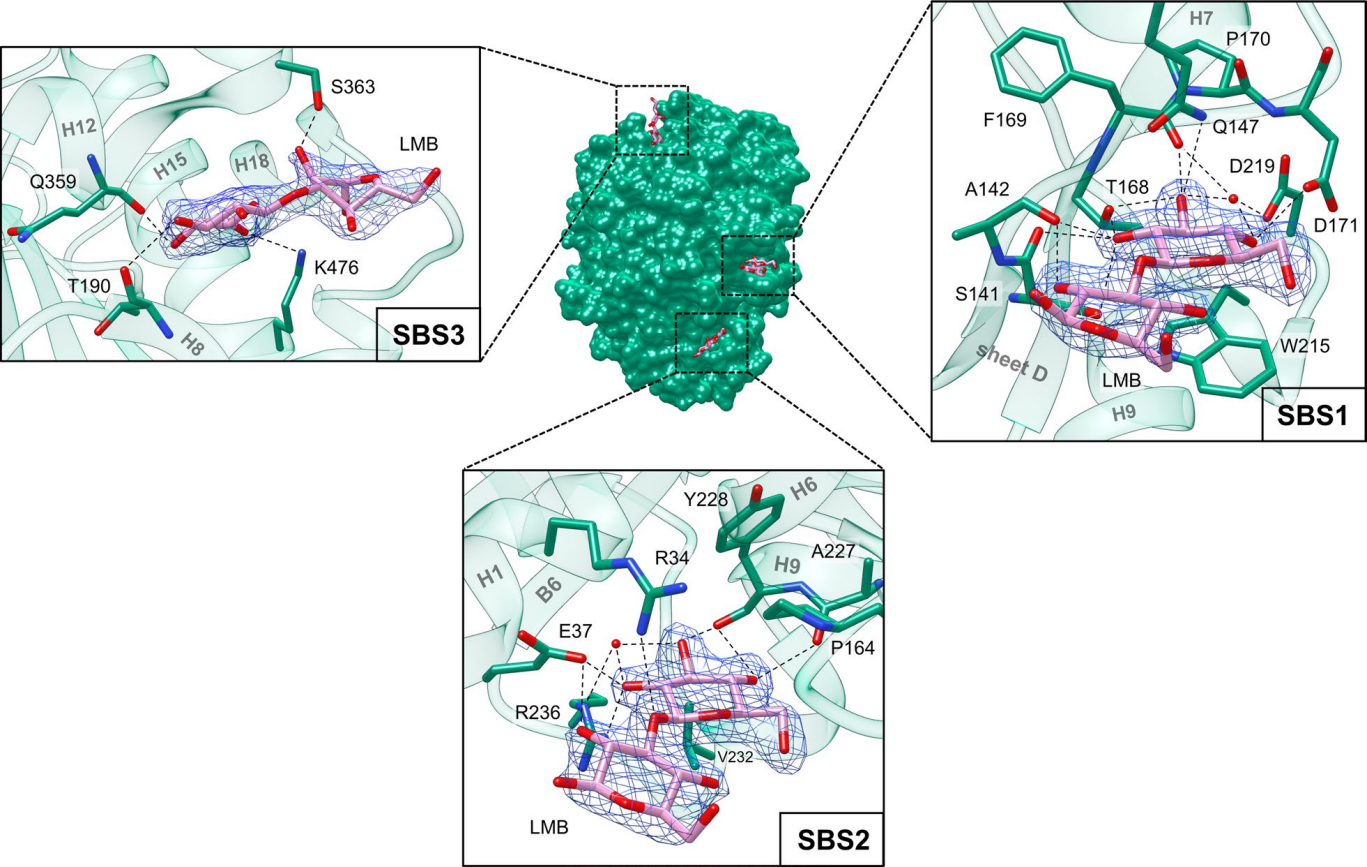
External sugar-binding sites (SBSs) in ODH-G3G. All three sites are located on the convex face of ODH, whose surface is represented in the center and colored in green. G3G molecules are represented in sticks and colored in pink (C atoms); O atoms are in red, N atoms in blue. The three sites are numbered according to their proximity to the active site entrance, located on the concave face of the protein (not shown). The three boxes show details of the interactions between protein and sugars. Water molecules are depicted as red spheres. Hydrogen bonds (distance < 3.2 Å) are represented with dotted lines. The 2F_o_-F_c_ electron density map (blue) is also depicted around G3G molecules and contoured at 1σ

A total of four G3G molecules are found in the structure of ODH-G3G: one in the active site (Fig. 6 and see Additional file 1: Fig. S8) and three on the protein external surface (Fig. 7). Similarly, the structure of ODH-GLC shows seven GLC molecules bound to the protein: four in the substrate-binding cavity (see Additional file 1: Fig. S10) and three on the protein surface (see Additional file 1: Fig. S11). In the case of ODH-GLC an additional GLC molecule (not shown) was identified at the interface between symmetry-related ODH molecules. This GLC ligand does not occupy a cavity and no G3G was found in the corresponding region of ODH-G3G, and it is likely to participate only to crystal contacts. Two GLC molecules closely mimic the binding of G3G to ODH active site: one (GLC1) is oriented as G3G reducing end, the other (GLC2) as the disaccharide non-reducing end pyranose (Fig. 6 and see Additional file 1: Fig. S8). Interestingly, in ODH-GLC two additional GLC molecules (GLC3 and 4) bind at the periphery (entrance) of the substrate-binding cavity and they could map a substrate diffusion pathway from the bulk to the FAD, suggesting that the external periphery of the substrate-binding tunnel might act as a funnel conveying substrate molecules to the reaction center (see Additional file 1: Fig. S10).

The electron density maps in the vicinity of the FAD cofactor accounting for bound G3G and GLC1 suggested that both anomeric forms of the saccharides bind to ODH active site. As such, we refined both the α and the β anomers with 35% and 65% occupancy, respectively, for both G3G and GLC1. The G3G and GLC1 β anomers are oriented in such a way that the reactive sugar O1 hydroxyl is hydrogen bonded to the N5 atom of the cofactor and the Nε2 atom of His528 (Fig. 6 and see Additional file 1: Figs. S8 and S9), in a position compatible for proton abstraction as hypothesized in the catalytic mechanism [18–22]. For both substrates, β anomers are also stabilized by hydrogen bonds with Val526 backbone carbonyl (O2 hydroxyl), with Gln331 side chain (O2 and O3 hydroxyls) and with the O4F atom of the pyrimidine portion of FAD (O6 hydroxyl). Apart from the latter, all hydrogen bonds with ODH are lost for G3G and GLC1 α anomers (see Additional file 1: Fig. S9), that are slightly shifted compared to the corresponding β anomers (Fig. 6 and see Additional file 1: Fig. S8). Notably, α anomer O1 hydroxyls do not interact with the catalytic histidine and the cofactor (see Additional file 1: Fig. S9), suggesting preferential ODH activity on β anomers, as confirmed by kinetics on GLC (see Additional file 1: Fig. S5). Although an additional hydrogen bond links the non-reducing pyranose of α-G3G, as well as GLC2, to the side chain of Asp418 (Fig. 6 and Additional file 1: Fig. S8), this bond is lost for bound β-G3G. Altogether these data suggest that hydrogen bonds do not play a major role in substrate binding and recognition (for more details see Additional file 2: Sect. 2).

Instead, both G3G and GLC α and the β anomers are stabilized by CH-π interactions, established between aromatic π-systems (protein sidechains) and pyranose C–H bonds. The two side chains of Trp430 and Phe416 lie almost on the same plane, forming an aromatic platform above the β-face of G3G reducing end and GLC1 (Fig. 6, and see Additional file 1: Fig. S8). These two amino acids account for the binding of both G3G and GLC anomers (see Additional file 1: Fig. S9), and possibly discriminate between the two anomers (for more details see Additional file 2: Sect. 3). This aromatic platform is extended also above the β-face of G3G non-reducing unit. The disaccharide end and GLC2 are in fact engaged in CH-π interactions with Phe421, belonging to the substrate-binding loop (see Additional file 1: Fig. S9). Phe421 phenyl ring lies parallel to the non-reducing pyranose ring (Fig. 6 and see Additional file 1: Fig. S8). On the other side of ligands, the aromatic ring of Tyr64 stabilizes the α faces of G3G glucosyl units and GLC in both anomeric forms (Fig. 6 and see Additional file 1: Fig. S8) by van der Waals interaction, and by one polar contact with the β anomer reducing end (see Additional file 1: Fig. S9). Despite it was proposed that Tyr64 may bind GLC in *Af*GDH through canonical hydrogen bonds between substrate and tyrosine hydroxyls [20], in the light of the results reported herein and previously, the role of this tyrosine residue might be more puzzling to decipher (for more details see Additional file 2: Sect. 4).

To summarize, structural analysis of ODH revealed two main contributions to substrate binding in the proximity of the cofactor: (i) CH-π stabilization due to electron-rich aromatic residues (Phe416, Phe421 and Trp430), with two well-defined pyranose binding sites that also explain ODH preference for G3G over GLC (for more details see Additional file 2: Sect. 3); and (ii) the steric effects of these and other hydrophobic residues, including Tyr64, that impose a directionality to G3G and GLC molecules within a V-shaped tunnel close to FAD, which provides the perfect environment to bind β(1→3) oligosaccharides (GLC1 and 2, Fig. 6 and see Additional file 1: Fig. S10).

### Structures of ODH bound to sugars: external sugar-binding sites

A peculiar feature of sugar-bound ODH structures is the presence of three sugar-binding sites (SBSs) on the external protein convex side, distant from the active site entrance, located on the concave side (Fig. 7). Their position and the details of the sugar–protein interactions are shown for ODH-G3G in Fig. 7 (see Additional file 1: Fig. S11 for ODH-GLC). G3G molecules bind to these sites through their non-reducing units, mainly contributing to SBSs interactions, while the reducing ones are exposed to the bulk and do not seem to participate to SBSs binding. GLC molecules bind in the same fashion and are superimposable to the non-reducing units of G3G. Both ligands show well-defined electron densities and B-factors comparable to those of the surrounding protein atoms, suggesting a tight binding.

SBS1 is a small pocket with residues from β-sheet D, from helix H7 and from the protruding loop which connects B5 and H9. Most of the sugar–protein contacts form a dense network of hydrogen bonds involving sugar hydroxyl groups: the O2 atom is stabilized by Ser141 sidechain hydroxyl and by Ser141 and Ala142 backbone carbonyls; the O3 atom by Thr168 and Gln147 side chains, by the carbonyl group of Phe169 and by Asp219 side chain through a water molecule; finally, the O4 atom interacts with Asp219 though the same water molecule and to Asp171 sidechain. The reducing unit of G3G does not contribute significantly to binding, as it only interacts with Ala142 peptide carbonyl group through the O2 hydroxyl. The sugar molecules in SBS1 are further stabilized by the aromatic side chain of Trp215, engaged in CH-π interactions with the α-face of the sugar ring.

SBS2 is located between H1 and H9, right below β-sheet D. As in SBS1, several interactions between the sugar molecules and the protein environment could be identified: GLC O2 atom is stabilized by hydrogen bonds with Glu37, Arg236 and a water molecule; the O3 atom also binds to Arg236 through the same water molecule, and to Tyr228 backbone carbonyl; the O4 hydroxyl group is involved in hydrogen bonds with the two peptide carbonyl groups of Tyr228 and Ala227. In the case of G3G, an additional hydrogen bond between the oxygen atom of the G3G β(1→3) glycosidic bond and the side chain of Arg34 is observed. As in SBS1, the reducing glycosyl unit of G3G provides a minor contribution to ligand binding, in this case through a hydrogen bond between the O2 atom and Glu37. Unlike in SBS1, no CH-π stabilization was observed in SBS2.

SBS1 and SBS2 are close to each other, while SBS3 is further away from the active site entrance and is located between helices H8 and H12. Sugars in SBS3 show higher temperature factors (B-factors) than in SBS1 and 2 and interact with the protein through three hydrogen bonds: the O3 and O4 sugar atoms interact with the peptide carbonyl group of Thr190 and Gln359, respectively, while the O6 atom interacts with Lys476 side chain. In the case of G3G, one hydrogen bond is also found between the O2 atom of G3G reducing end and Ser363 side chain and backbone amide.

## Discussion

### Role of ODH in lignocellulose breakdown

**Fig. 8:**
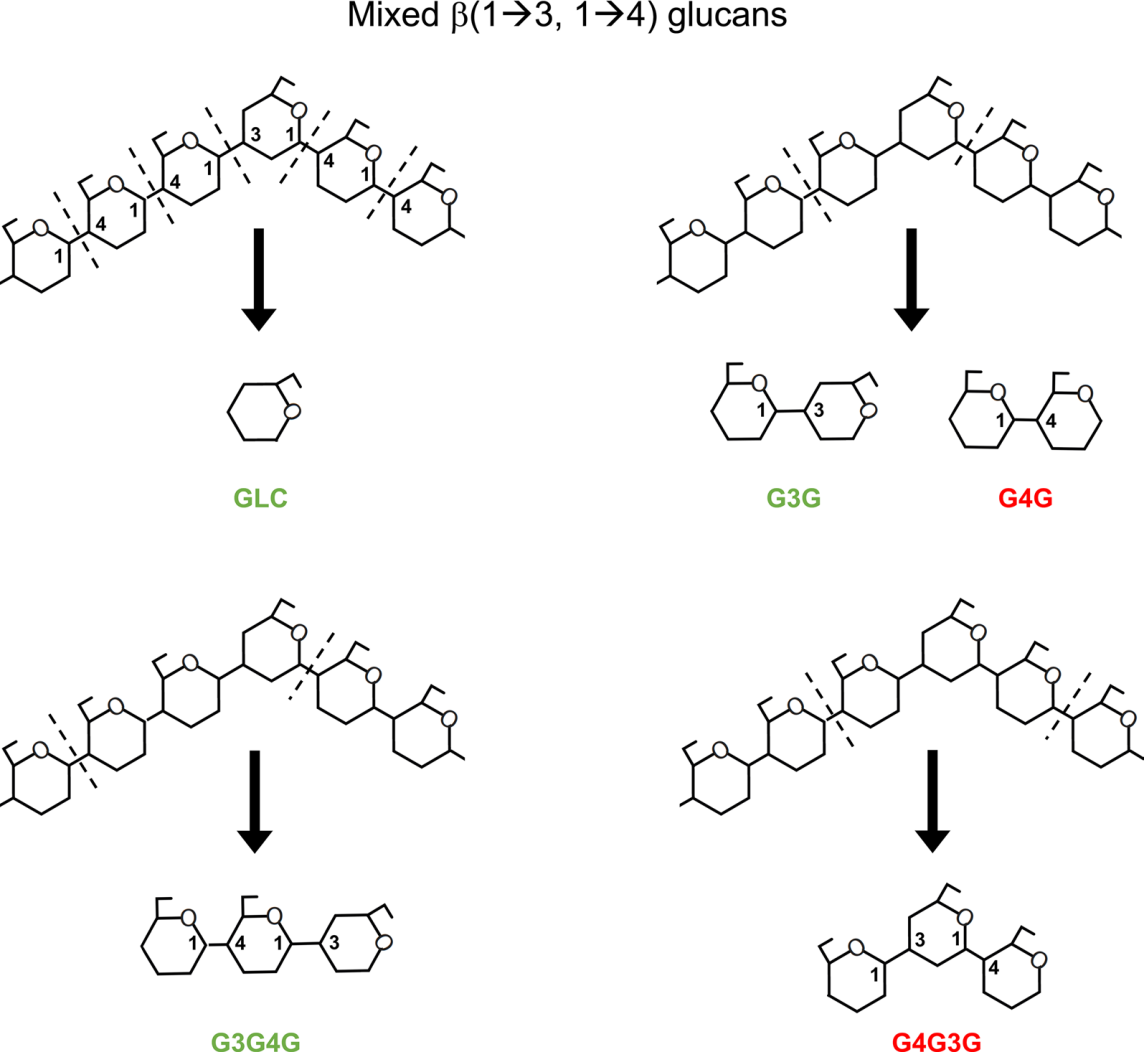
Schematic representation of the possible cleavage products of mixed β(1→3, 1→4) glucans. Depending on the type of cleavage performed by lytic enzymes, saccharides with different types of glycosidic bonds can be released. ODH is able to oxidize GLC, G3G and G3G4G (in green), but not active on G4G and G4G3G (in red). Some carbon atoms of glucosyl units in substrate and cleavage products are labeled

Functional data show that the optimal substrates of ODH are sugars endowed with a β(1→3) glycosidic bond next to their reducing glucosyl unit, with G3G being the preferred substrate among the compounds tested. This β(1→3) glycosidic bond is found in β(1→3) glucans, major components of the fungal cell wall [27], in bacterial curdlan [28], as well as in β(1→3) callose and in mixed β(1→3, 1→4) glucans typically present in the plant cell wall. Mixed glucans are part of the hemicellulose matrix and assures plant cell wall growth and integrity together with cellulose, pectin, lignin and other compounds [29, 30]. Like other plant cell wall components, hemicelluloses are fully degraded by fungi and other organisms. As for cellulose, this is achieved through the action of glycoside hydrolases (GHs), such as enzymes belonging to the GH12 and GH45 CAZy families [31], which show endo-glucanase activity toward mixed β(1→3, 1→4) glucans. Despite the enzymatic specificity in terms of glycosidic linkage, we observed that ODH is able to accept electrons from oligosaccharides displaying different degrees of polymerization, provided that a β(1→4) follows a β(1→3) glycosidic bond, counting from the reducing-end of the oxidizable saccharide. In the light of these observations, we can speculate on the physiological role of ODH. Among all possible oligosaccharides released by the cleavage of mixed β(1→3, 1→4) glucans, ODH is able to oxidize GLC, G3G and G3G4G (Fig. 8). G4G also produced in large amount by fungal cellulolysis, cannot be oxidized by ODH but by the flavodomain (CAZy subfamily AA3_1) of cellobiose dehydrogenase (CDH). Electrons deriving from G4G oxidation are used to fuel lytic polysaccharide monooxygenase (LPMO) activity [32]. Completion of the LPMO catalytic cycle requires in fact a Cu(II) reduction step, that can also be sustained in vitro by diffusible redox mediators which are reduced by AA3_2 dehydrogenases, such as ODH [33, 34]. AA3_2 dehydrogenases might also reduce phenoxy radicals and assist laccases (CAZy family AA1) in lignin degradation [13, 14], whereas AA3_2 oxidases would produce hydrogen peroxide, a co-substrate for LPMOs and fungal peroxidases (CAZy family AA2) [35, 36]. Many other enzymes play similar roles in fungal lignocellulose breakdown. A notable example is AA7 flavoenzymes from Ascomycetes, which have been shown to oxidase cellulose-derived oligosaccharides [37], hemicellulose-derived xylo-oligosaccharides [38] and even polysaccharides, including β-glucans [39]. Within this scenario, the identification of ODH activity points towards another source of oligosaccharides, containing β(1→3) glycosidic bonds and derived from hemicellulose (β-glucans), within the palette of compounds that fungi can oxidize to maintain redox homeostasis of lignocellulose-degrading enzymes. These observations can be interpreted as an enzymatic adaptation to substrate availability and they expand the range of known sugars derived from hemicelluloses that can be oxidized by Basidiomycetes. Activity on oligosaccharides is not unique to ODH, as it has also been reported for CDH (AA3_1) and AA7 enzymes, although regarding different substrates, which, in either cases, allows the oxidation of only partially deconstructed polysaccharide. Finally, the GOX/GDH clade of AA3_2 enzymes seems to include enzymes with different physiological functions: monosaccharide oxidoreductases, such as GOX and GDH from Ascomycetes, and oligosaccharide oxidoreductases, such as ODH, that seem more efficient in transforming plant cell wall components, as expected for saprophytic and pathogenic Basidiomycetes.

### Structure–function analysis of ODH

The three crystallographic structures herein reported, provide a molecular explanation for ODH substrate specificity. Preferred activity towards a disaccharide is structurally justified by the presence of two pyranose binding pockets, relying, respectively, on Phe416/Trp430, close to the FAD cofactor, for the reducing saccharide end, and on Phe421, that stabilizes the non-reducing glucosyl unit, owing to the conformational change of the substrate-binding loop. Interestingly, this rearrangement occurs also when GLC binds to ODH active site, where two molecules of GLC mimic the disaccharide (G3G) binding geometry. Finally, we can not exclude that the substrate-binding loop might also play a role in escorting monosaccharides toward the active site and, indeed, the presence in ODH-GLC of four GLC molecules bound to ODH active site and its funnel-shaped opening suggests the possible existence of a well-defined substrate diffusion pathway.

The main forces responsible for G3G and GLC orientation in ODH active site are CH-π interactions with aromatic residues (Phe416, Phe421 and Trp430), which are rather conserved in ODH-related enzymes. On the contrary, in GDH class-I and GOX enzymes, such as *Af*GDH (PDB: 4YNT [20]) and *An*GOX (1CF3 [19]), the molecular determinants for the formation of the sugar–enzyme complex rely on specific hydrogen bonds between sugar hydroxyl groups and conserved polar residues from the active site (see Additional file 1: Figs. S6 and S9).

To our knowledge, the presence of secondary SBSs on the enzyme surface has not yet been reported for other GMC oxidoreductases. However, they seem to be conserved to a certain extent within the GOX/GDH clade, particularly SBS1 and 2 (see Additional file 1: Fig. S6). The molecular bases of sugar recognition in these sites restrict the hypothetical natural binders to a limited group of carbohydrates. Only monosaccharides and oligosaccharide non-reducing ends bound to the SBSs in ODH crystallographic structures. Potential, naturally occurring ligands may be found among non-reducing ends of polysaccharides, like cellulose or hemicellulose, and of their cleavage products. Interestingly, branched polysaccharides in hemicellulose, such as xyloglucans, glucuronoxylans, galactomannan and galactoglucomannans, are plausible binders too. The xylose, glucuronic acid and galactose units, forming the branches of these polysaccharides, possess in fact free O2, O3 and O4 hydroxyl groups [29] with the same stereochemistry found in GLC, except for the galactose O4 hydroxyl group. If the external SBSs identified in ODH are able to recognize branched polysaccharides, they might have a physiological role, as they may anchor ODH and possibly other AA3 enzymes to specific polysaccharide domains of lignocellulose where their catalytic activity is needed.

## Conclusions

We identified oligosaccharides that contain β(1→3) glycosidic bonds, such as laminarin (G3G), as preferred enzyme substrates, and we provided an explanation for enzyme promiscuity towards GLC. Indeed, ODH activity on monosaccharides previously led to the definition of GDH class-III enzymes. However, in the light of the results described herein we renamed the investigated enzyme oligosaccharide dehydrogenase (ODH). The ability to accept electrons from mono- and oligosaccharides deriving from hemicellulose breakdown is consistent with ODH being an efficient enzymatic tool for saprophytic and pathogenic Basidiomycetes, which need to master lignocellulose deconstruction for their survival.

The structure of ODH, the first characterized enzyme within the ODH subclade of AA3_2 enzymes, represents a major step toward understanding the enzymatic diversity within the GOX/GDH clade as it represents the first structure of such an enzyme derived from a Basidiomycete and the second structure of a dehydrogenase from the GOX/GDH clade. Structure–function analysis points out some novel, unexpected features of ODH substrate recognition, especially: (i) the lack of specific glucose-binding residues (as found instead in GDH class-I and GOX) and the presence of three key aromatic residues (Phe416, Phe421 and Trp430 in ODH) providing CH-π stabilization; (ii) the involvement of a highly flexible substrate-binding loop in the process of substrate recognition; (iii) the presence of external SBSs, which might play a role in sensing substrate availability and directing the enzymatic activity toward the polysaccharide matrix, thus contributing to the overall enzymatic efficiency. ODH characterization is a good example of extended versatility, even within a small group of phylogenetically related enzymes such as the GOX/GDH clade of AA3_2 enzymes. As seen from previous phylogenetic analysis, increased biodiversity supported by novel, uncharacterized and unexpected enzymatic functions is yet to be expected within the AA3_2 subfamily of GMC oxidoreductases, including about 10 phylogenetic (sub)clades of enzymes of unknown function.

Auxiliary enzymes involved in lignocellulose degradation, like ODH, represent promising candidates for the development of novel biotechnologies in the context of the plant biomass biorefinery. Indeed, ODH could fuel LPMO enzymatic activity and inactivate laccase-generated phenoxy radicals, improving as such the breakdown of recalcitrant cellulose and inhibiting lignin repolymerization during biomass enzymatic saccharification. The preference of ODH for oligosaccharides containing β(1→3) glycosidic bonds implies that ODH and related enzymes might be particularly useful and effective in treating specific types of biomass. This is the case for plants that are naturally rich in mixed β(1→3, 1→4) glucans, such as those belonging to the *Poales* order, which includes extensively cultivated plant species (wheat, rice and corn, among others) and accounts for a large fraction of poorly valorized agro-industrial residues.

## Methods

### Chemicals

2,6-Dichlorophenolindophenol (DCIP or DCPIP), GLC, GAL, XYL, LAC, MAN, MAL and G4G were purchased from MilliporeSigma (Burlington, MA, USA). G3G, G3G3G, G3G3G3G, G3G4G, G4G3G, G4G4G, G4G4G4G were purchased from Megazyme (Bray, Ireland).

### Crystallization and crystal handling

ODH was expressed and purified as described in Piumi et al*.* [14]. Purified ODH was exchanged, after IMAC Ni–NTA affinity chromatography, in buffer with no imidazole and concentrated to 17.4 mg mL^−1^ for crystallization trials. ODH concentration was measured using a NanoDrop™ 2000 (Thermo Fisher Scientific, Waltham, MA, USA), assuming an extinction coefficient ε_280_ = 89,840 M^−1^ cm^−1^ (Abs_280_ 0.1% = 1.510) estimated from ODH amino acid sequence using ExPASy Protparam [40]. Crystallization plates were initially set up automatically using a Crystal Phenix robot (Art Robbins Instruments, Sunnyvale, CA, USA) following the sitting-drop vapor-diffusion method at 294 K using 96-well Intelli-plates (Art Robbins Instruments, Sunnyvale, CA, USA). Single crystals were obtained using the AmSO4 suite (Qiagen, Hilden, Germany) crystallization screen. The reproducibility of crystallization conditions was tested setting up hand-made plates using the hanging-drop vapor-diffusion method at 294 K, which led to the formation of larger crystals. Well-diffracting single crystals grew by mixing 1 μL of protein solution and 1 μL of reservoir solution containing 2 M (NH_4_)_2_SO_4_ and equilibrating the droplet against 0.5 mL of reservoir solution. Cryoprotection was achieved by transferring ligand-free ODH crystals in a solution containing 2 M (NH_4_)_2_SO_4_ and 2 M LiSO_4_, which were then flash-frozen in liquid nitrogen. ODH-GLC crystals were prepared by soaking ligand-free ODH crystals in a solution containing 2 M (NH_4_)_2_SO_4_ and 80% GLC which also acted as cryoprotectant; after about 12 min incubation, crystals were flash-frozen in liquid nitrogen. Analogously, ODH-G3G crystals were obtained from 20 min soaking in a solution containing 2 M (NH_4_)_2_SO_4_ and 8% G3G. Crystals were cryoprotected in 2 M (NH_4_)_2_SO_4_ and 2 M LiSO_4_, and flash-frozen in liquid nitrogen prior to data collection at synchrotron radiation sources. Similar soaking experiments with cellobiose were performed too. Data collected on these crystals did not show any electron density due to G4G binding in the active site, despite the ligand was clearly bound to (and modeled at) the three SBSs (data not shown).

### Structure determination and refinement

X-ray diffraction data of ligand-free ODH and ODH-G3G were collected at the Diamond synchrotron (Harwell, UK), beamline I24; data for ODH-GLC were collected at ELETTRA (Trieste, Italy), beamline XRD2. All datasets were collected at 100 K using a PILATUS detector. Data were indexed, scaled and integrated using the XDS package [41]. Molecular replacement was carried out using MOLREP [42] from the CCP4 suite [43]. The structure of *Af*GDH at 1.78 Å resolution (PDB entry 4YNT [20], 36.4% sequence identity to ODH) was used as the search model to calculate the initial crystallographic phases of ligand-free ODH, whose structure was then employed to obtain the initial phases for both ODH-GLC and ODH-G3G. Iterative structure refinement and model building were carried out using REFMAC5 [44] and COOT [45], respectively, both implemented in the CCP4 suite. The Translation–Libration–Screw-rotation (TLS) model of rigid-body harmonic displacements was included during the last cycles of automated refinement [46]. 5% of the reflections were excluded from refinement for cross validation by means of the free R-factor [47]. Manual model building was performed based on the F_o_-F_c_ map contoured at 3σ and the 2F_o_-F_c_ map at 1σ. Validation of the models, including Ramachandran statistics and B-factor analysis, was carried out using the Multimetric model geometry validation tool implemented in the CCP4 suite. Protein sequence alignment was performed using Clustal Omega [48], and structural superposition using Superpose [49], implemented in the CCP4 suite. Molecular graphics figures were produced using Chimera [50].

### Spectrophotometric assays for enzymatic activity

Functional assays have been carried out using DCIP as the secondary electron acceptor in the half-reaction responsible for FAD oxidation [14]. Enzymatic activity was evaluated spectrophotometrically monitoring the loss in DCIP absorbance upon reduction at 520 nm (ΔAbs_520_, ε_520_ = 6800 M^−1^ cm^−1^), which represents a pH-independent isosbestic point [51]. In agreement with what previously reported [14], experiments carried out in the presence of GLC did not indicate significant electron transfer to dioxygen, and no ODH-generated H_2_O_2_ could be observed in the pH range 3–9, that we tested using 2,2′-azino-bis(3-ethylbenzothiazoline-6-sulfonate) (ABTS) and horseradish peroxidase (HRP) as previously described [13]. This confirms that the enzyme acts as a dehydrogenase and not an oxidase.

All spectrophotometric assays were performed using a sample final volume of 0.1 mL in a 0.2 mL 96-well plate (Corning Costar, Corning, NY, USA) using a Multiskan GO microplate spectrophotometer (Thermo Scientific, Waltham, MA, USA) in static (substrate screening) and in kinetic (enzyme kinetics) modes. Plates and solutions without the enzyme were first equilibrated at 303.15 K (30 °C); upon addition of ODH, solutions were automatically mixed and the signal at 520 nm was recorded. Each measurement was taken at least in triplicate independent experiments. The absorbance signal of DCIP was converted into concentration units by comparison to a calibration curve (Abs_520_ *vs* [DCIP]) measured concomitantly to each experiment. The slope of a linear fit of Abs_520_ *vs* [DCIP] was used to calculate Δ[DCIP] in all experiments.

The pH-dependence of ODH enzymatic activity was assessed using 720 mM GLC and 0.4 mM DCIP either in 50 mM citrate–phosphate buffer pH 3.5, 4.5, 5.5, 6.5 and 7.5, or in 50 mM Tris·HCl pH 8.5. To start the reaction ODH was added at a final concentration of 39.7 nM. Initial velocities were measured spectroscopically by monitoring the rate of the absorbance decay at 520 nm over time for a total of 20 min. Initial rates were estimated by applying a linear fit to the linear region of the time trace. Optimal pH fell in the range 5.5–6.0, as previously reported [12]. All remaining spectrophotometric assays were carried out in 0.4 mM DCIP and 50 mM citrate–phosphate buffer pH 5.5.

ODH substrate screening was performed using fourteen different sugar compounds: GLC, XYL, GAL, MAN, LAC, MAL, G4G, G3G, G4G4G, G3G3G, G4G3G, G3G4G, G4G4G4G and G3G3G3G (see Additional file 1: Fig. S1), as well as on sophorose, gentiobiose, nigerose, kojibiose and isomaltose (see Additional file 1: Fig. S2). Because of different solubility, all sugar substrates were employed at a fixed concentration of 2.5 mM, with the exception of GLC that was also used at 250 mM as a positive control of ODH reaction. The enzyme was added at a final concentration of 39.7 nM to start the reaction (*t* = 0). DCIP reduction was followed over time by acquiring triplicates of the Abs_520_ after an incubation of 2 and 5 min, as well as 3 and 19 h. Each data point was collected on three identical, independently prepared reaction mixtures.

The same experimental setup was used to evaluate the kinetic parameters of ODH using GLC and G3G as substrates. GLC and G3G were tested at different concentrations ranging from 0 to 3000 mM and from 0 to 115.5 mM, respectively. For each substrate concentration initial velocities were measured spectroscopically by monitoring the rate of absorbance decay at 520 nm over time for a total of 20 min in triplicate experiments. Initial rates were estimated by fitting the linear region of the time trace. Initial velocities measured at various substrate concentrations were fitted to the hyperbolic equation of Michaelis–Menten, which was applied to either all concentration points (G3G) or to points that looked unaffected by inhibition at extremely high substrate concentrations (GLC), as better detailed in the Results section. All data were fitted using the Kaleidagraph software package.

### Analysis of ODH reaction products by LC–MS

Liquid Chromatography was performed on a UHPLC Ultimate 3000RS (Thermo Scientific) coupled to a Charged Aerosol Detector (CAD Corona, Thermo Scientific) and an ISQ-EM mass spectrometer with heated ESI-interface (Thermo Scientific). The eluent was split 1:1 and the resulting flow from the LC to the MS was in all cases 0.125 mL/min. The heated ESI was operated at 348 K in negative mode at − 2 kV spray current, with a sheath gas flow of 23.5 and an auxiliary gas flow of 2.6 (arbitrary units). The capillary temperature was 523 K. UHPLC-ESI–MS data were acquired and analyzed with the Chromeleon software v7.2.10 (Thermo Scientific). An Acquity UHPLC BEH Amide column (2.1 mm × 150 mm, 1.7 mm, Waters, Milford, USA) was used for chromatographic separation of analytes. ODH enzymatic assays were performed on GLC and G3G in order to detect and identify the enzymatic products. Reaction mixtures were prepared in unbuffered water and contained 37.5 mM substrate, 45 mM DCIP and 90 nM ODH. Reactions were run for 21 h at 348 K. Enzymatic assay aliquots were diluted 5 times in acetonitrile (20 µL enzymatic assay + 80 µL acetonitrile) and 2µL of the diluted samples were injected. The column temperature was maintained at 30 °C. The isocratic elution method used ammonium formate 12 mM-acetonitrile (35%/65% v/v) at a flow rate of 0.25 mL min^−1^. The mass range from 50 to 1500 m/z was monitored.

## Supplementary Information

**Additional file 1**: Supplementary Figures.

**Additional file 2**: Key amino acids and interactions: a more detailed analysis.

## Data Availability

The datasets used and/or analyzed during the current study are available from the corresponding author on reasonable request. Coordinates and mtz files for the crystallographic structures have been deposited in the Protein Data Bank (PDB) with accession codes 6XUT (ligand-free ODH), 6XUU (ODH-GLC) and 6XUV (ODH-G3G).

## Abbreviations

*Af*GDH: Glucose dehydrogenase from *A. flavus*
*An*GOX: Glucose oxidase from *A. niger*
CAZy: Carbohydrate-active enzymes database
DCIP: 2,6-Dichlorophenolindophenol
FAD: Flavin adenine dinucleotide
G3G: Laminaribiose
G3G3G: Laminaritriose
G3G3G3G: Laminaritetraose
G3G4G: 1,3;1,4 β-Glucotriose B
G4G: Cellobiose
G4G3G: 1,3;1,4 β-Glucotriose A
G4G4G: Cellotriose
G4G4G4G: Cellotetraose
GAL: D-galactose
GDH: Glucose dehydrogenase
GLC: D-Glucose
GMC: Glucose-methanol-choline
GOX: Glucose oxidase
LAC: D-Lactose
LGC: D-Glucono-δ-lactone
MAL: D-Maltose
MAN: D-Mannose
ODH: Oligosaccharide dehydrogenase
ODH-G3G: Structure of ODH in complex with laminaribiose (PDB: 6XUV)
ODH-GLC: Structure of ODH in complex with glucose (PDB: 6XUU)
PDB: Protein data bank
r.m.s.d.: Root-mean-square deviation
SBS: Sugar-binding site
XYL: D-Xylose

## References

[1] Janusz G, Pawlik A, Sulej J, Swiderska-Burek U, Jarosz-Wilkołazka A, Paszczynski A (2017). Lignin degradation: microorganisms, enzymes involved, genomes analysis and evolution. FEMS Microbiol Rev.

[2] Kirk TK, Farrell RL (1987). Enzymatic “combustion”: the microbial degradation of lignin. Annu Rev Microbiol.

[3] Levasseur A, Drula E, Lombard V, Coutinho PM, Henrissat B (2013). Expansion of the enzymatic repertoire of the CAZy database to integrate auxiliary redox enzymes. Biotechnol Biofuels.

[4] Cantarel BL, Coutinho PM, Rancurel C, Bernard T, Lombard V, Henrissat B (2009). The Carbohydrate-Active EnZymes database (CAZy): an expert resource for Glycogenomics. Nucleic Acids Res.

[5] Levasseur A (2014). The genome of the white-rot fungus *Pycnoporus cinnabarinus*: a basidiomycete model with a versatile arsenal for lignocellulosic biomass breakdown. BMC Genomics.

[6] Lomascolo A, Uzan-Boukhris E, Herpoël-Gimbert I, Sigoillot J, Lesage-Meessen L (2011). Peculiarities of *Pycnoporus* species for applications in biotechnology. Appl Microbiol and Biotechnol.

[7] Estrada Alvarado I, Navarro D, Record E, Asther M, Asther M, Lesage-Meessen L (2003). Fungal biotransformation of p-coumaric acid into caffeic acid by *Pycnoporus cinnabarinus*: an alternative for producing a strong natural antioxidant. World J Microbiol Biotechnol.

[8] Tilay A, Bule M, Annapure U (2010). Production of biovanillin by one-step biotransformation using fungus *Pycnoporous cinnabarinus*. J Agric Food Chem.

[9] Gross B, Yonnet G, Picque D, Brunerie P, Corrieu G, Asther M (1990). Production of methylanthranilate by the basidiomycete *Pycnoporus cinnabarinus*. Appl Microbiol and Biotechnol.

[10] Cao Y, Chen SS, Zhang S, Ok YS, Matsagar BM, Wu KC (2019). Advances in lignin valorization towards bio-based chemicals and fuels: lignin biorefinery. Bioresour Technol.

[11] Shekher R, Sehgal S, Kamthania M, Kumar A (2011). Laccase: microbial sources, production, purification, and potential biotechnological applications. Enzyme Res.

[12] Sygmund C, Klausberger M, Felice AK, Ludwig R (2011). Reduction of quinones and phenoxy radicals by extracellular glucose dehydrogenase from *Glomerella cingulata* suggests a role in plant pathogenicity. Microbiology.

[13] Mathieu Y, Piumi F, Valli R, Aramburu JC, Ferreira P, Faulds CB (2016). Activities of secreted aryl alcohol quinone oxidoreductases from *Pycnoporus cinnabarinus* provide insights into fungal degradation of plant biomass. Appl Environ Microbiol.

[14] Piumi F, Levasseur A, Navarro D, Zhou S, Mathieu Y, Ropartz D (2014). A novel glucose dehydrogenase from the white-rot fungus *Pycnoporus cinnabarinus*: production in *Aspergillus niger* and physicochemical characterization of the recombinant enzyme. Appl Microbiol Biotechnol.

[15] Cavener DR (1992). GMC oxidoreductases. A newly defined family of homologous proteins with diverse catalytic activities. J Mol Biol.

[16] Sützl L, Foley G, Gillam EMJ, Bodén M, Haltrich D (2019). The GMC superfamily of oxidoreductases revisited: analysis and evolution of fungal GMC oxidoreductases. Biotechnol Biofuels.

[17] Sützl L, Laurent CVFP, Abrera AT, Schütz G, Ludwig R, Haltrich D (2018). Multiplicity of enzymatic functions in the CAZy AA3 family. Appl Microbiol Biotechnol.

[18] Hecht HJ, Kalisz HM, Hendle J, Schmid RD, Schomburg D (1993). Crystal structure of glucose oxidase from *Aspergillus niger* refined at 2.3 Å resolution. J Mol Biol.

[19] Wohlfahrt G, Witt S, Hendle J, Schomburg D, Kalisz HM, Hecht HJ (1999). 18 and 19 Å resolution structures of the *Penicillium amagasakiense* and *Aspergillus niger* glucose oxidases as a basis for modeling substrate complexes. Acta Crystallogr D Biol Crystallogr.

[20] Yoshida H, Sakai G, Mori K, Kojima K, Kamitori S, Sode K (2015). Structural analysis of fungus-derived FAD glucose dehydrogenase. Sci Rep.

[21] Leskovac V, Trivić S, Wohlfahrt G, Kandrac J, Pericin D (2005). Glucose oxidase from *Aspergillus niger*: the mechanism of action with molecular oxygen, quinones, and one-electron acceptors. Int J Biochem Cell Biol.

[22] Wohlfahrt G, Trivić S, Zeremski J, Pericin D, Leskovac V (2004). The chemical mechanism of action of glucose oxidase from *Aspergillus niger*. Mol Cell Biochem.

[23] Ferri S, Kojima K, Sode K (2011). Review of glucose oxidases and glucose dehydrogenases: a bird’s view of glucose sensing enzymes. J Diabetes Sci Technol.

[24] Garcia-Rubio LH (1992). Refractive index effects on the absorption spectra of macromolecules. Macromolecules.

[25] Sampedro JG, Muñoz-Clares RA, Uribe S (2002). Trehalose-mediated inhibition of the plasma membrane H+-ATPase from Kluyveromyces lactis: dependence on viscosity and temperature. J Bacteriol.

[26] Lyubimov AY, Heard K, Tang H, Sampson NS, Vrielink A (2007). Distortion of flavin geometry is linked to ligand binding in cholesterol oxidase. Protein Sci.

[27] Gow NAR, Latge JP, Munro CA (2017). The fungal cell wall: structure, biosynthesis, and function. Microbiol Spectr.

[28] McIntosh M, Stone BS, Stanisich VA (2005). Curdlan and other bacterial (1–>3)-beta-D-glucans. Appl Microbiol Biotechnol.

[29] Scheller HV, Ulvskov P (2010). Hemicelluloses. Annu Rev Plant Biol.

[30] Buckeridge MS, Rayon C, Urbanowicz B, Tiné MAS, Carpita NC (2004). Mixed linkage (1—>3), (1—>4)-β-D-glucans of grasses. Cereal Chem.

[31] Payne CP, Knott BC, Mayes HB, Hansson H, Himmel ME, Sandgren M (2015). Fungal cellulases. Chem Rev.

[32] Tan TC, Kracher D, Gandini R, Sygmund C, Kittl R, Haltrich D (2015). Structural basis for cellobiose dehydrogenase action during oxidative cellulose degradation. Nat Commun.

[33] Kracher D, Scheiblbrandner S, Felice AKG, Breslmayr E, Preims M, Ludwicka K (2016). Extracellular electron transfer systems fuel cellulose oxidative degradation. Science.

[34] Garajova S, Mathieu Y, Beccia MR, Bennati-Granier C, Biaso F, Fanuel M (2016). Single-domain flavoenzymes trigger lytic polysaccharide monooxygenases for oxidative degradation of cellulose. Sci Rep.

[35] Hangasky JA, Iavarone AT, Marletta MA (2018). Reactivity of O2 versus H2O2 with polysaccharide monooxygenases. PNAS.

[36] Bissaro B, Várnai A, Røhr ÅK, Eijsink VGH (2018). Oxidoreductases and reactive oxygen species in conversion of lignocellulosic biomass. Microbiol Mol Biol Rev.

[37] Lee M-H, Lai W-L, Lin S-F, Hsu C-S, Liaw S-H, Tsai H-C (2005). Structural characterization of glucooligosaccharide oxidase from *Acremonium strictum*. Appl Environ Microbiol.

[38] Vuong TV, Vesterinen A-H, Foumani M, Juvonen M, Seppälä J, Tenkanen M (2013). Xylo- and cello-oligosaccharide oxidation by gluco-oligosaccharide oxidase from Sarocladium strictum and variants with reduced substrate inhibition. Biotechnol Biofuels.

[39] Foumani M, Vuong TV, MacCormick B, Master ER (2015). Enhanced polysaccharide binding and activity on linear β-glucans through addition of carbohydrate-binding modules to either terminus of a glucooligosaccharide oxidase. PLoS ONE.

[40] Wilkins MR, Gasteiger E, Bairoch A, Sanchez JC, Williams KL, Appel RD, Hochstrasser DF (1999). Protein identification and analysis tools in the ExPASy server. Methods Mol Biol.

[41] Kabsch W (2010). XDS. Acta Crystallogr D Biol Crystallogr.

[42] Vagin A, Teplyakov A (1997). MOLREP: an automated program for molecular replacement. J Appl Crystallogr.

[43] Collaborative Computational Project 4. The CCP4 suite: programs for protein crystallography. Acta Crystallogr D Biol Crystallogr 1994;50:760–3. 10.1107/S0907444994003112 15299374

[44] Pannu N, Murshudov G, Dodson E, Read RJ (1998). Incorporation of prior phase information strengthens maximum-likelihood structure refinement. Acta Crystallogr D Biol Crystallogr.

[45] Emsley P, Lohkamp B, Scott WG, Cowtan K (2010). Features and development of Coot. Acta Crystallogr D Biol Crystallogr.

[46] Winn M, Isupov M, Murshudov GN (2000). Use of TLS parameters to model anisotropic displacements in macromolecular refinement. Acta Crystallogr D Biol Crystallogr.

[47] Brunger AT (1992). Free R value: a novel statistical quantity for assessing the accuracy of crystal structures. Nature.

[48] Madeira F, Park YM, Lee J, Buso N, Gur T, Madhusoodanan N (2019). The EMBL-EBI search and sequence analysis tools APIs in 2019. Nucleic Acids Res.

[49] Krissinel E, Henrick K (2004). Secondary-structure matching (SSM), a new tool for fast protein structure alignment in three dimensions. Acta Crystallogr D Biol Crystallogr.

[50] Pettersen EF, Goddard TD, Huang CC, Couch GS, Greenblatt DM, Meng EC (2004). UCSF Chimera—a visualization system for exploratory research and analysis. J Comput Chem.

[51] Armstrong JM (1964). The molar extinction coefficient of 2,6-dichlorophenol indophenol. Biochim Biophys Acta.

