# Supplementary material for: Crystal structure and functional characterization of an oligosaccharide dehydrogenase from Pycnoporus cinnabarinus provides insights into fungal breakdown of lignocellulose
Source: Biotechnol Biofuels. 2021 Jul 22;14:161. doi: 10.1186/s13068-021-02003-y (PMC8296622; doi:10.1186/s13068-021-02003-y)

Additional File 1. Supplementary figures

Figure S1. Chemical structures of the monosaccharides and gluco-oligosaccharides tested for ODH activity. Carbon atoms involved in glycosidic bonds are numbered. D-galactose and D-mannose are epimers of D-glucose, respectively at C4 and C2. D-xylose differs from D-glucose for the absence of the group CH_2-OH at C5. D-lactose is a disaccharide composed by D-galactose linked to D-glucose by a β(1→4) glycosidic bond and D-maltose is a disaccharide made by two units of glucose joined with an α(1→4) bond.

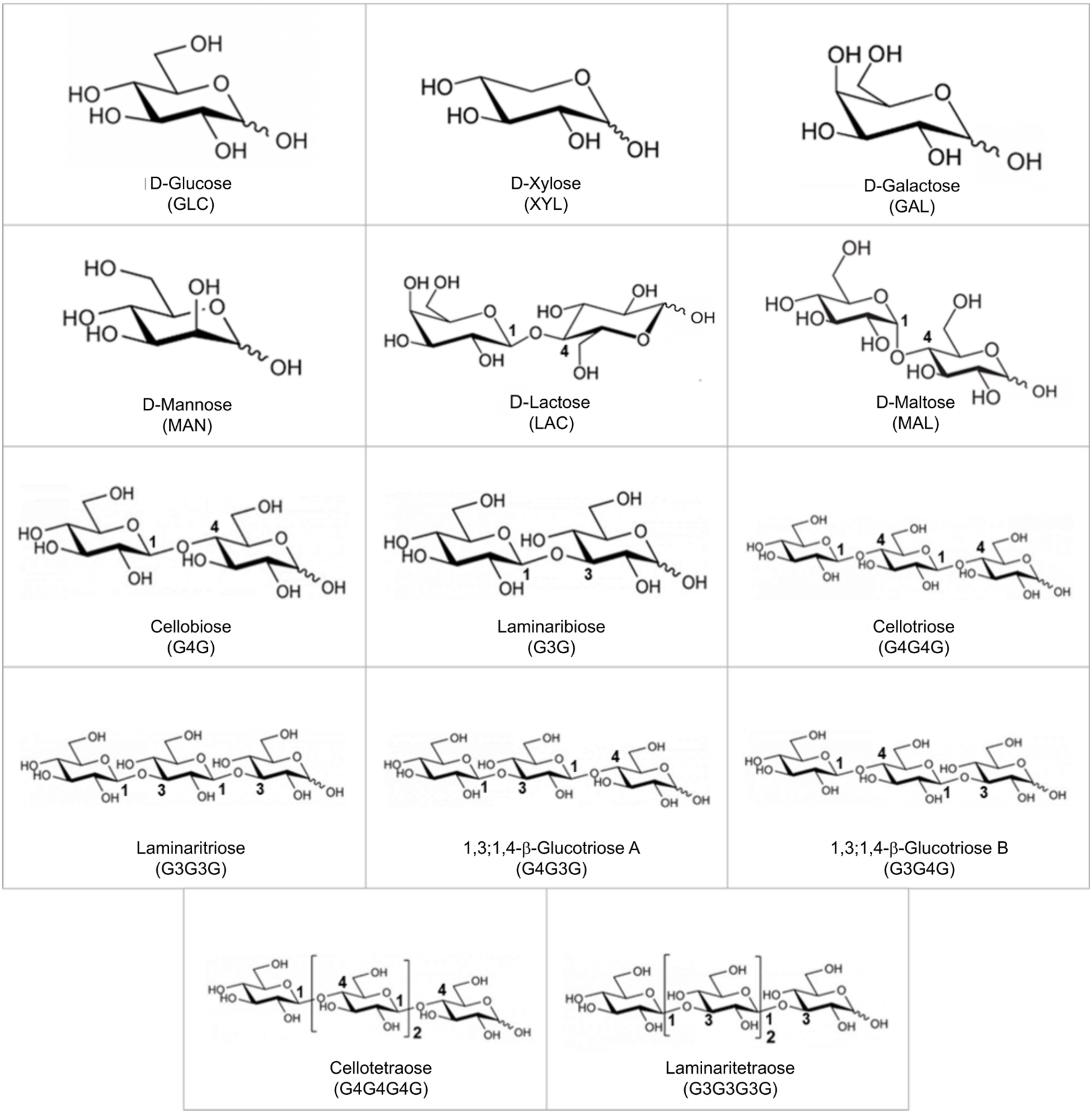

Figure S2. Extended ODH substrate screening. To evaluate ODH activity towards all glucobiose isomers with a reducing end, substrate screening was further extended to sophorose, gentiobiose, nigerose, kojibiose and isomaltose, whose structures are represented within the squared boxes. The experiment was conducted as described for obtaining the data reported in Fig. 2 of the main text. Enzyme activity, comparable to the one observed on GLC, was only detected for gentiobiose. To exclude any possible artifacts, we verified by LC/MS that gentiobiose was not hydrolyzed (data not shown).

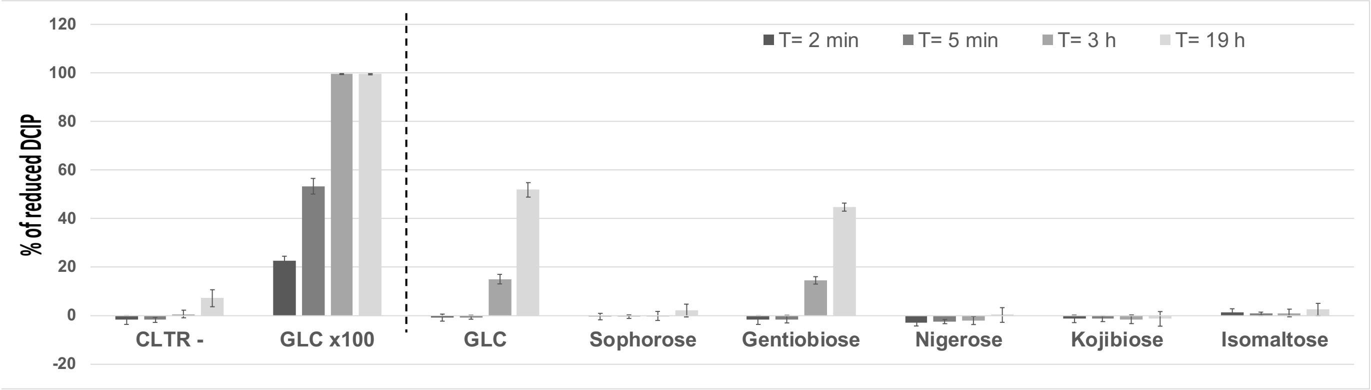

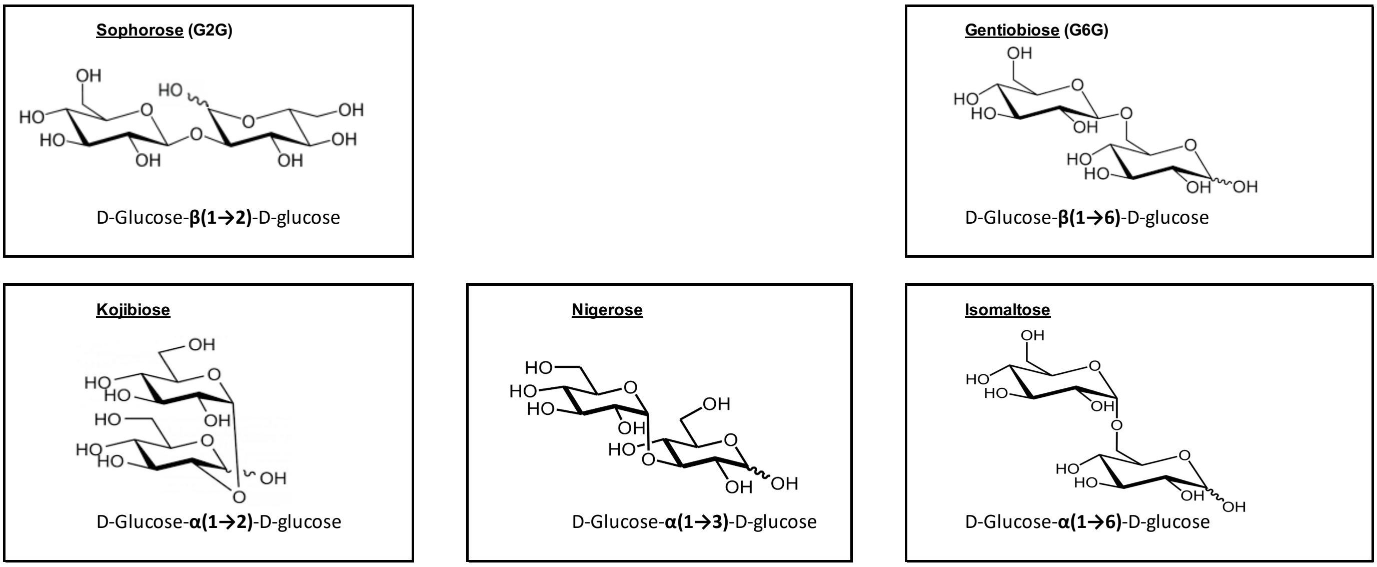

Figure S3. LC-MS analysis of enzymatic products. Top panel: chromatografic separation of G3G (grey) and of an overnight G3G/DCIP/ODH reaction (black). G3G (MW=342 g/mol) appeared as a double chromatografic peak (peak 1) corresponding to the α- and β-anomers, as confirmed by the detection of ions having m/z values of 341 [M-H], 387 [M+Formate -H] and 683 [2M-H] (not shown). After enzyme oxidation only (peak 2), laminaribionic acid (MW=358 g/mol) was detected by ions having m/z values of 357 [M-H] and 715 [2M-H] (bottom panel), while no formate adduct was detected. DCIP (MW=268 g/mol, oxidized) was also detectected at an m/z value of 266 (and a minor peak at 268 for reduced DCIP). The chemical nature of laminaribionic acid confirms specific enzymatic oxidation of the C1 hydroxyl of G3G reducing end, as oxidation of other G3G hydroxyls would not result into hydrolysable enzyme products. The same enzymatic reaction (not shown) was assessed for GLC (MW=180 g/mol), detected by ions having an m/z value of 225 [M+Formate -H], and gluconic acid (MW=196 g/mol), detected at an m/z value of 195 [M-H] in GLC/DCIP/ODH overnight incubations, in agreement with what we previously reported [14].

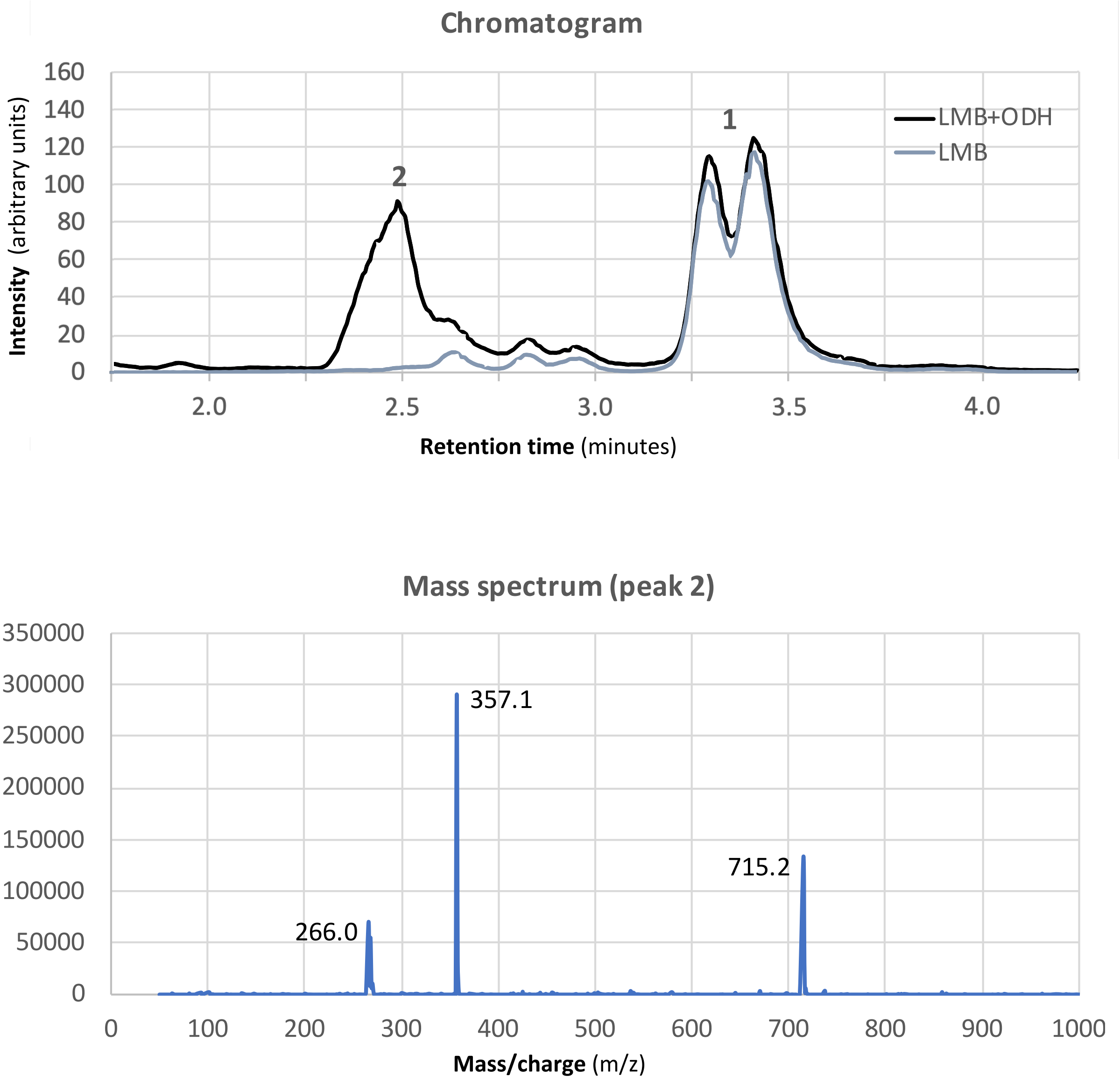

Figure S4. Full-range enzymatic kinetics of ODH on GLC. Initial rates of DCIP reduction as a function of GLC concentration, ranging between 0 and 3000 mM. The solid line represents a fit to the equation: $v_0 = (V'_{max} \times [S])/(K'_M + [S] + [S]^2/K_i)$, which takes substrate inhibition into account. In the equation v_0 is the initial velocity of the reaction, V'_{max} is the maximum velocity of the reaction corresponding to substrate saturation of the enzyme, K'_M is the Michaelis–Menten constant, [S] is the substrate analytical concentration and K_i corresponds to the dissociation constant for substrate binding to the enzyme. The fit is poor, suggesting that other inhibition models should be taken into account.

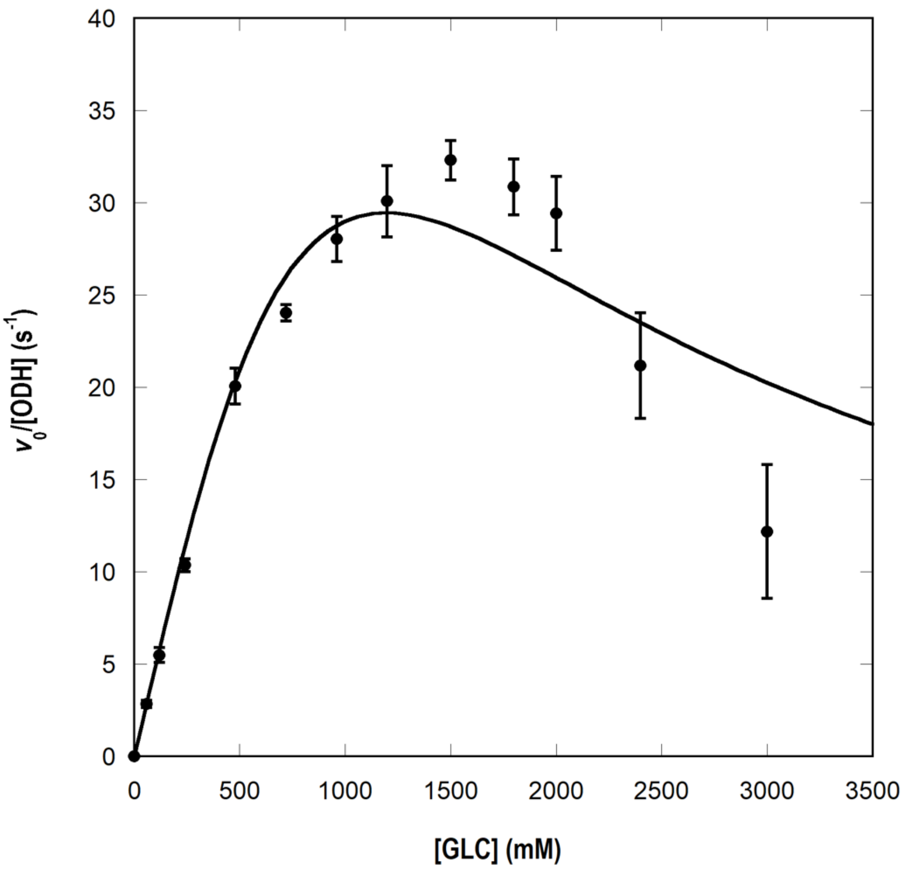

Figure S5. Comparison of ODH activity on different GLC anomers. Initial rates of DCIP reduction were measured on a 96-well plate as described in Methods (Spectrophotometric assays for enzymatic activity), with 25 mM of either freshly dissolved α-GLC (grey empty points) or preequilibrated GLC solutions (mixtures of α- and β-anomers, black solid points). Solid lines represent a fit to the Abs_{520} decrease (initial velocity), and the error bars the standard deviation of three independent replicas of the reaction mixtures. Measurements start 3-4 minutes after dissolving α-GLC in 30° C water, accounting for the time required to: dissolve α-GLC, add it to the reaction mixtures, dispense ODH to trigger the reaction and run data collection. Within the observed time window, ODH activity is twice faster on preequilibrated GLC (both anomers) than on freshly dissolved α-GLC.

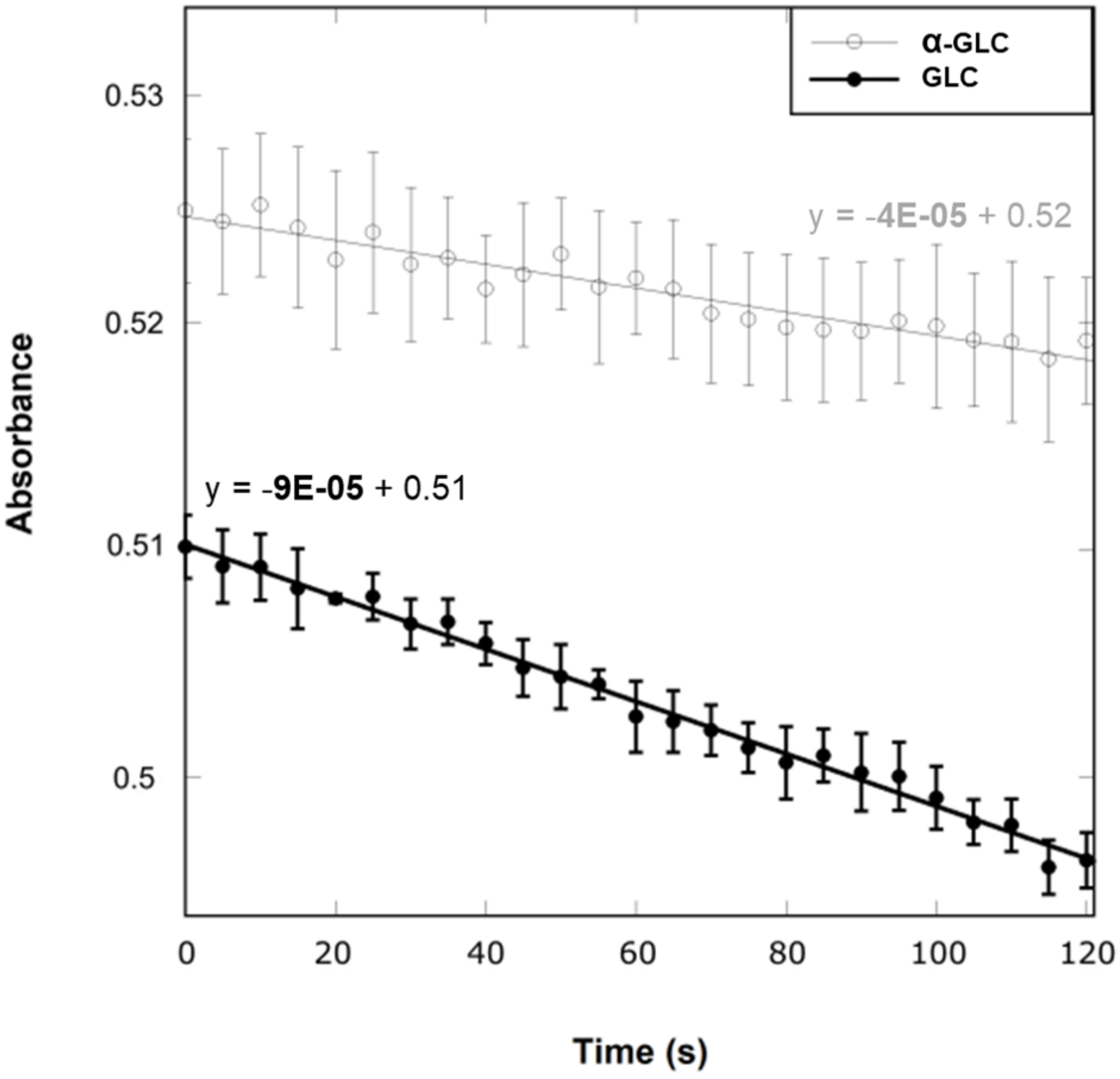

Figure S6. Multiple sequence alignment of ODH with enzymes from the GOX/GDH clade. Four equally distant genes per each previously defined subclade of the GOX/GDH clade [16] were used for Multiple Sequence Alignment (MSA). In the preliminary Table, subclade nomenclature, coloring scheme and order are as in [16], apart for the suggested renaming of the GDH III subclade into ODH. GOX-like and ODH-like subclades correspond to poorly defined groups that appear to be phylogenetically close to the GOX and the ODH subclades, respectively [16]. MSA analysis includes protein sequences from both Ascomycota (white background) and Basidiomycota (grey), corresponding to either characterized enzymes (bold) or to hypothetical genes from genomic data. Genes are named by their Uniprot identifier, preceded by a four letter PDB code if the three-dimensional structure is known, and by a GOX or GDH label for assigned functions. In the MSA, ODH amino acid sequence is in bold. The following coloring has been used to identify conserved residues involved in: substrate binding / catalysis / FAD ring distortion (blue); SBS1 (orange), SBS2 (yellow) and SBS3 (green); GLC-binding (H-bonds) in GOX/GDH enzymes (purple), and their loss in ODH (purple framed, golden box); G3G and GLC binding (H-bonds and CH-π interactions) in ODH (red); and substrate-binding loop and related residues (magenta).

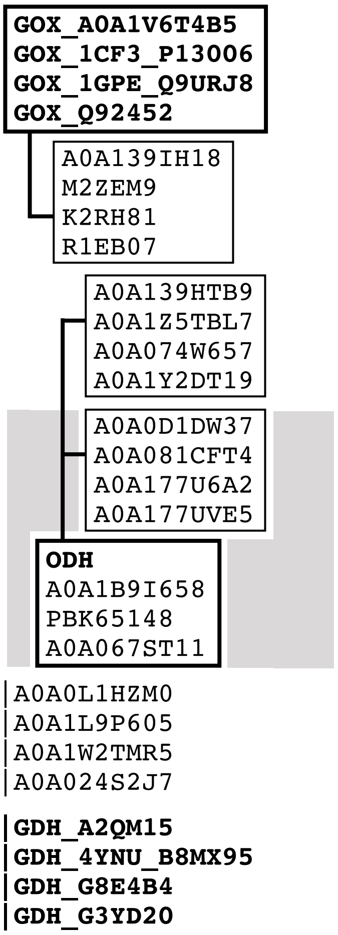

Subclade	Phylum	Organism
GOX	ASCO	*Penicillium flavigenum*<br>*Aspergillus niger*<br>*Penicillium amagasakiense*<br>*Talaromyces flavus*
GOX -like	ASCO	*Pseudocercospora musae*<br>*Pseudocercospora fijiensis*<br>*Macrophomina phaseolina*<br>*Neofusicoccum parvum* UCRNP2
ODH -like	ASCO	*Mycosphaerella eumusae*<br>*Hortaea werneckii* EXF-2000<br>*Aureobasidium namibiae* CBS 147.97<br>*Pseudomassariella vexata*
ODH -like	BASI DIO	*Ustilago maydis* 521<br>*Moesziomyces antarcticus*<br>*Tilletia caries*<br>*Tilletia caries*
ODH (GDH III)	BASI DIO	*Pycnoporus cinnabarinus*<br>*Kwoniella pini* CBS 10737<br>*Armillaria solidipes*<br>*Galerina marginata* CBS 339.88
GDH II	ASCO	*Stemphylium lycopersici*<br>*Aspergillus versicolor* CBS 583.65<br>*Rosellinia necatrix*<br>*Trichoderma reesei* RUT C-30
GDH I	ASCO	*Aspergillus niger* CBS 513.88<br>*Aspergillus flavus* NRRL3357<br>*Colletotrichum gloeosporoides*<br>*Aspergillus niger* ATCC_1015

```
|GOX_A0A1V6T4B5      SDPTKVEGKTYDYVIAGGGLTGLTVAAKLSENPKIKVLVIEKGFYESNDGPIIEDP----  88
|GOX_1CF3_P13006     TDPKDVSGRTVDYIIAGGGLTGLTTAARLTENPNISVLVIESGSYESDRGPIIEDL----  87
|GOX_1GPE_Q9URJ8     SDPSKVAGKTYDYIIAGGGLTGLTVAAKLTENPKIKVLVIEKGFYESNDGAIIEDP----  88
|GOX_Q92452          SDPSKVAGKTYDYIIAGGGLTGLTVAAKLTENPKIKVLVIEKGFYESNDGAIIEDP----  88

   |A0A139IH18       ---TAQLKAEYDYIICGGGTAGLAVANRLSADSKNSVLVVEAGING--------------  60
   |M2ZEM9           ---TAQLKKEYDYIVCGGGTAGLAVANRLSADPKNSVLVVEAGING--------------  60
   |K2RH81           ---SSQLLESYDYIICGGGTSGLTVANRLTELADATVLVIEAGPESIS------------  65
   |R1EB07           QPQGSDLLDSYDYIICGGGTSGLTVASRLSELADATVLVIEAGSDT--------------  71

     |A0A139HTB9     SSASGIANASFDYVVCGGGLTGLTIASRLSEDPSVSVLVIEAGYDNHTS-PLVNDV----  92
     |A0A1Z5TBL7     TDVNAVADKSFDYVICGGGLTGLTVASRLSEDPDISVLVIEAGYDNHND-PRVYDV---- 104
     |A0A074W657     RASNMSANNSFDYVVVGGGLTGLTVASRLSENPTIRVLVIEDGQDNHND-PRVSDV----  74
     |A0A1Y2DT19     HDASVVSNQTFDYIIAGGGVTGLTVASKLSEDPSIRVLVVEGGSNDWDD-AMVHDI----  85

     |A0A0D1DW37     TDASKISNNKYDYIIVGGGLAGLTVANRLSANSAVKVLVIEAGNDERAN-PDVYNV----  81
     |A0A081CFT4     TDANSLSGRSYDYIIVGGGLAGLTVANRLSANSSTSVLVIEAGNDDRNN-ADVYNV----  80
     |A0A177U6A2     TDPSIATSRTFDYIIVGGGLAGLVVANRLSEKANINVLVIEAGADTRND-DRIASL----  99
     |A0A177UVE5     TDPAVATGQVWDYVIVGGGLAGLTVANRLTENSGISVLVIEAGADTRDD-FRIRSL----  90

  |ODH               SDPTVVNGQTYDYIVVGGGLTGTTVAARLAENSSLQILMIEAGGDDRTN-PQIYDI----  61
  |A0A1B9I658        TDPSQVSNQTFDYVIVGGGLGGLVVANRLSENPDITVLVIEAGSDNRDD-PRVYDP----  81
  |PBK65148          TDASQANAQSFDYIVVGAGLTGTTVAARLAEDSGVTVLLIEAGADNRDD-SRVYDI----  83
  |A0A067ST11        TSASLANGQTFDYIVVGAGLTGTTVAARLAEDPTVTVLLIETGADNRND-SRVYDI----  86

|A0A0L1HZM0          TVCATCASQSFHYVIVGGGPAGLLVANRLSADPNTTVAVIEAGDFAYNN-PNVTSVPKSI  71
|A0A1L9P605          ASNAGANTNRFDFVIIGGGTAGLAVASRLSEIPDITIAVIEAGKDEGNN-PNVSSV----  65
|A0A1W2TMR5          AMAGAARAETFDYIIAGAGTCGLVLANRLSADPAVRVAVIEPGEDVRNN-ANVTDP----  76
|A0A024S2J7          PQVLASSTDSYDYIIVGAGTCGLLLANRLSQDANYTVAIIDPGADERDN-PNVVDP----  74

|GDH_A2QM15          PSTRL-CGPQYDYIVVGGGTSGLVVANRLSENPNVSVLIIEAGGSVLNN-SNVTDV----  75
|GDH_4YNU_B8MX95     PAGRAKNTTTYDYIVVGGGTSGLVVANRLSENPDVSVLLLEAGASVFNN-PDVTNA----  72
|GDH_G8E4B4          APRDQAAATAYDYIVIGGGTSGLVVANRLSEDASVSVLVIEAGDSVLNN-ANVTNA----  75
|GDH_G3YD20          -KSHADSPAHYDFVIVGGGTSGLVVANRLSELSDVTVAVIEAGESALNN-FNVSNV----  71
                                 .::: *.*   *    *  :*:        :  ::: *


|GOX_A0A1V6T4B5      NAYGEI--FG---TSVDQNYLTVPLI--NNRTGEIKSGLGLGGSTLINGDSWTRPDKVQI 141
|GOX_1CF3_P13006     NAYGDI--FG---SSVDHAYETVELA-TNNQTALIRSGNGLGGSTLVNGGTWTRPHKAQV 141
|GOX_1GPE_Q9URJ8     NAYGQI--FG---TTVDQNYLTVPLI--NNRTNNIKAGKGLGGSTLINGDSWTRPDKVQI 141
|GOX_Q92452          NAYGQI--FG---TTVDQNYLTVPLI--NNRTNNIKAGKGLGGSTLINGDSWTRPDKVQI 141

   |A0A139IH18       --------------TTAYWGYHSLPQKYANGRIQDLPAGKTVGGSSQINGRVYSRPDRREI 107
   |M2ZEM9           --------------TTAYWNYHSLPQKYANGRIQDLPAGKTVGGSSQINGRVYSRPDRRSI 107
   |K2RH81           ---------SQ---SQGDWRYPNGPERFASGRSQTLPAGKVLGGSSVINGQVYLRAEQAQI 114
   |R1EB07           --------------TTANWNYPSGPERFASGRSQTLPAGKALGGSSAINGRVYLRAEQSQI 118

     |A0A139HTB9     RTYGLA--FN---TSLDHQIHSTPVEWQDGERLLLVTGKTLGGSGSLNGASWTKGAKSQY 147
     |A0A1Z5TBL7     RTYGEA--FE---SELDWNITSTPVPWQNDTGLLLVAGRTLGGSGSINGASWTKGPASQY 159
     |A0A074W657     RTYGQA--FE---SDLDYNMTSTPIPWRNGTKLPMAAGKTLGGSGSISGASWTKGPRTQY 129
     |A0A1Y2DT19     RTYGQA--FG---TYLDYNLTSTPISWRNGETLPMIAGHTLGGSGSINGASWTKAPKSQY 140

     |A0A0D1DW37     DNYGKA--FG---NDLTFKFNTVPQV--GGRTKAPLGGRTLGGSTSINGAAWNRASRAQY 134
     |A0A081CFT4     DNYGKA--FG---TDLTWHFTTVPQI--GGRTKAPLGGKTLGGSTSINGAAWNRASRAQY 133
     |A0A177U6A2     DAYGSV--FLSSSKDINWQFTTTN-------GKTISAGRGLGGSTSINGGAITRADTAQL 150
     |A0A177UVE5     DQYGKG--FAANNTDINWRYTATN-------GKLVSAGKGLGGSTSINGAAWTRGDVVEL 141

  |ODH               YEYGAV--FN---GPLDWAWEADQ-------GKVIHGGKTLGGSSSINGAAWTRGLNAQY 109
  |A0A1B9I658        YQYSVA--FN---TELDWNWPSSQ-------GRYIKGGKTLGGSTSINGLAQTRGQKAQY 129
  |PBK65148          YAYSQA--FG---TELDWSWSTDD-------GKTIHGGKTLGGSSSINGGHYTRGLDAQF 131
  |A0A067ST11        YEYGDA--FG---SELTYSWSADQ-------GKSILGGKTLGGSSSINGAAYTRGLNAQY 134

|A0A0L1HZM0          LEFGLF--IG---TSIDWGYTTVPQKYAGNKTLAYWAGKALGGSTTINGMTYLRAETDQI 126
|A0A1L9P605          VEYGGTAGQG---TQIDWSYETVEQRYAGGRKIGFHAGKAWGGTSTINGMTYIRAEEAQI 122
|A0A1W2TMR5          ANFTVP--YG---TSIDWQYSTTPQPGAGNRTLDWHAGKAIGGTSTINGMTYIRGDKAQF 131
|A0A024S2J7          LGWLGL--TG---TSVYWNYSSIPQEKLGGRVLEYDAGRGIGGTSLINGMTYIRGDKAQF 129

|GDH_A2QM15          NGYGLA--FG---TDIDWQYETINQSYAGDAPQVLRAGKALSGTSAINGMAYTRAEDVQV 130
|GDH_4YNU_B8MX95     NGYGLA--FG---SAIDWQYQSINQSYAGGKQQVLRAGKALGGTSTINGMAYTRAEDVQI 127
|GDH_G8E4B4          NGYGLA--FG---TDIDYAYQTTAQTYANNASTTLRAAKALGGTSTINGMAYTRAEASQI 130
|GDH_G3YD20          MGYSTA--FG---TEVDWAYQTENQTYAGGLQQTIRAGKALGGTSTINGMSYTRAEDVQI 126
                                                      .    .*:  :.*     :     .
```

```
|GOX_A0A1V6T4B5      DSWEKVFGM----EGWNWDNVFQYMQKAERSRPPTAA-QIEAGHFYDPACHGTDGTVHAG  196
|GOX_1CF3_P13006     DSWETVFGN----EGWNWDNVAAYSLQAERARAPNAK-QIAAGHYFNASCHGVNGTVHAG  196
|GOX_1GPE_Q9URJ8     DSWEKVFGM----EGWNWDNMFEYMKKAEAARTPTAA-QLAAGHSFNATCHGTNGTVQSG  196
|GOX_Q92452          DSWEKVFGM----EGWNWDSMFEYMKKAEAARAPTAA-QLAAGHYFNATCHGTNGTVQSG  196

   |A0A139IH18       DDWGQVNN-----AGWSWETLFPFYKSSEGLRPPTKQQQKDLENTYNPDYHGVAGPVNIT  162
   |M2ZEM9           DDWGNVNN-----AGWSWEKLFPFYKASEGLQPPTKQQQRDLENKYNPDYHGTAGPVNIT  162
   |K2RH81           DDWERAGN-----TGWNWATLFPYYLKSEQFAVPNER-QTDAGLTYTAEFHGTNGPVNTG  168
   |R1EB07           DDWEKAGN-----TGWNWEALWPYYLKSEQITAPNER-QTDGGLTYDPDFHGTDGPVNTG  172

     |A0A139HTB9     DLLPLLSGD----ESWDWASFNQYMLKAENFHRPSDSQRDVKGAHYESGFHGYSGPVQVS  203
     |A0A1Z5TBL7     DVLPLLTGD----DSWGWEGLNEYMLIAENFNLPEPR-EIASGARYDRAYHGQGGPVEVS  214
     |A0A074W657     DLLPLLTGD----ESWGWDGLNQYMLGAEHFYAPSNE-LKSKGADYDGHKHGFSGPVQVS  184
     |A0A1Y2DT19     DLLPLVTGD----ESWAFDSFNEIMLSAEKFTPPTQA-LTSKGANYVPAYHGTNGNVSVS  195

     |A0A0D1DW37     DALGALINS--ADAGWNWNGLLGYMKKSENFVAPNQD-QRNLGAKWDPSVHGTSGPLEIG  191
     |A0A081CFT4     DALGGLVGS----SGWSWDGLLGYMKKSENFIAPTSA-QQAAGARYDASVHGSGGPLEIG  188
     |A0A177U6A2     DNIGKLGN-----QNWDSNSLFQYMKKAETFVTPNTA-QVNAGARFTAEAHGTNGPLTIR  204
     |A0A177UVE5     DNIGRLGN-----TGWNYDSMLSYMNKAEKFTIPNGD-VASLGGQYNATSHGFSGYVSVR  195

  |ODH               DSWSSLLEPEEASVGWNWNNLFGYMKKAEAFSAPNDQ-QRAKGADSIASYHGTTGPVQAT  168
  |A0A1B9I658        DSLSTFLGGDDGNGYWNWDGMYFGMLKSEGFSAPNDQ-QKDAGASSNPAYHNTSGPLQVT  188
  |PBK65148          DAWSSLLEDYESDVGWNWDGMLNYMKKSEAFSAPNGQ-QSAKGAQFNGDYHGWSGPVQVT  190
  |A0A067ST11        DSWSALLEPSEASVGWNWQGLWDYMQKSETFSAPNSQ-QAAKGAQAISSYHGFSGPVRVT  193

|A0A0L1HZM0          DAWEDLGN-----PGWNWDTLWEYFLKQEGFQPPTDK-LEQRGAAYEVGAHGRDGELAVG  180
|A0A1L9P605          DSWETLGN-----KGWSWASLWPYHLKSERYETPTKA-QLAAGASYVDSYHGRDGPLNVA  176
|A0A1W2TMR5          DAWEALGA-----KGWNWETLYPYYKKAEKFTVPSDA-QAAAGATYDPEVHGDAGHVHTG  185
|A0A024S2J7          DAWEQLGN-----PGWNWTTMLKHFKKIEQFFPPLPW-QETAGALYEDEYHGTSGEMHVG  183

|GDH_A2QM15          DAWQTIGN-----EGWTWDSLFPYYRKSENLTAPTAS-QRARGATYDPSANGEEGPLSVA  184
|GDH_4YNU_B8MX95     DVWQKLGN-----EGWTWKDLLPYYLKSENLTAPTSS-QVAAGAAYNPAVNGKEGPLKVG  181
|GDH_G8E4B4          DAWETVGN-----EGWNWDALLPYYLKSETFQAPDAERSIKGHISYESDVHGHDGPLYTA  185
|GDH_G3YD20          DNWEVLGN-----DGWNWKNLFQYYKKSEGFQVPTKD-QIAHGASYNASYHGLNGPLKVG  180
                     *              *    .       *    *                :.  * :


|GOX_A0A1V6T4B5      PRD-------------------NGKPWSPLMRALMNT-VSAFGVPVQKDFHCGHPRGVSM  236
|GOX_1CF3_P13006     PRD-------------------TGDDYSPIVKALMSA-VEDRGVPTKKDFGCGDPHGVSM  236
|GOX_1GPE_Q9URJ8     ARD-------------------NGQPWSPIMKALMNT-VSALGVPVQQDFLCGHPRGVSM  236
|GOX_Q92452          ARD-------------------NGQPWSPIMKALMNT-VSALGVPVQQDFLCGHPRGVSM  236

   |A0A139IH18       YP--------------------AV-GVPEYFNILEDT-AKTFSIPRNRDFNGGDARGLST  200
   |M2ZEM9           YP--------------------AV-GVPDYFRLLEET-AKAFSIPRIRDFNGGDARGLST  200
   |K2RH81           WP--------------------TEVAIETYLNQLEEA-YASIGMPRIQDPNGGEMRGLST  207
   |R1EB07           WP--------------------AEASIETYLSQLEEA-YASIGMPRIDDPNGGNMRGLST  211

     |A0A139HTB9     FG--------------------AGMYGGTQLASLKASENAISNFTRNFDAASGQTNGGTI  243
     |A0A1Z5TBL7     FA--------------------AGIFEKPQQDALEASYKVWEGLERIDDAAAGEVNGATI  254
     |A0A074W657     FA--------------------QGMFGSIQQPALNASQQVW-GLEHVSDAASGKVNGATT  223
     |A0A1Y2DT19     FA--------------------QGIFSSIQLPAINASQMVWEGLEVAVDAASGEVNGVTT  235

     |A0A0D1DW37     FTQIRNNNRRSTTNVGGASQQWKRMFTGPQQPAFIKAVGETLGVQQVDDQCSGQANSVAF  251
     |A0A081CFT4     FSQIRNQRR-----SGGASQEWRRMFIGPQQAAFIAAIGETLGVQHVDDQCAGSANSVAY  243
     |A0A177U6A2     FGEVQSSSRRDLLSNSSSAQLEKRFYTGPYQSSFVQSVKTALGVDRINDLSDGHTNGVAY  264
     |A0A177UVE5     FGDVVKDNTTQR-RSSPSAGLHERFWTGPQQPAFVKAVDQTLGIKPLADVAAGKTNGVAY  254

  |ODH               FP--------------------DEMYGGPQMPAFVNTVVNVTGMPHYKDLNGGTPNCVSI  208
  |A0A1B9I658        YP--------------------DELFHGAQQKYFKEVVSTNFSVASSPDADDGNANVVAF  228
  |PBK65148          YP--------------------DEMYGGPQQGDFVNTIVNLTGIYHSPDLNGGGANSVSI  230
  |A0A067ST11        YP--------------------DDMYGGPQQLAFVDTIVALTGISHFKDLNGGTPNCVSI  233

|A0A0L1HZM0          FT--------------------PYLVGQGFADLLKTT-SEAMGIPYNQDPNDGVMRGFNT  219
|A0A1L9P605          YQ--------------------YGLQNGSFASLAKET-WEAFGMPFNPDVNGGNIRGFSV  215
|A0A1W2TMR5          FP--------------------FSLVNGSLHDLAVDT-WAALGYELNEEVNAGSVRGFGA  224
|A0A024S2J7          FN--------------------PALLSGSFYGDVKAS-WAALGQTLNRDASSGDTAGFDV  222

|GDH_A2QM15          WP--------------------DI-PANNLTNTLNAT-FQGLGVPWTEDVNGGKMRGFNV  222
|GDH_4YNU_B8MX95     WS--------------------GSLASGNLSVALNRT-FQAAGVPWVEDVNGGKMRGFNI  220
|GDH_G8E4B4          YA--------------------YGSTNDSYPTSLNAT-YQALNVPWKEDIAGGSMVGFAS  224
|GDH_G3YD20          WP--------------------NSMTNSSVFPVLEQT-FEKLGVQYNPDSEGGKMVGFTV  219
                                                                .     :   *
```

```
GOX_A0A1V6T4B5      IPNNL----HENQIRADAAREWLLPNY-QRDNLQILTGQKVGKVLFNQTA------SGPK  285
GOX_1CF3_P13006     FPNTL----HEDQVRSDAAREWLLPNY-QRPNLQVLTGQYVGKVLLSQNG------TTPR  285
GOX_1GPE_Q9URJ8     IMNNL----DENQVRVDAARAWLLPNY-QRSNLEILTGQMVGKVLFKQTA------SGPQ  285
GOX_Q92452          IMNNV----DENQVRVDAARAWLLPSY-QRPNLEILTGQMVGKVLFKQTA------SGPQ  285

A0A139IH18          YPGFFVINAQREQLRESAREAYYLPVV-GRPNLELLDETMCQRIDWENGS---G-GGDLK  255
M2ZEM9              YPGFFVINAQREQRRESSREAYYLPIV-GRTNLELIDETMCQRIDWEKSS---D-GGDLK  255
K2RH81              YPRTRQA-INDRDVRESAASAYYEPVR-DRTNLDVLLNTSVLRIVWAEND---DADGNAI  262
R1EB07              YPRTQQN-VNDQDVREDANTAYYQQVA-DRTNLDVLTNTSCLRIVWAESD---DADGNAI  266

A0A139HTB9          IPNMVVP--EENQNRSSPFTAYAQNQVQERNNFLILTGHRVTEIVWQETK----ERAPLV  297
A0A1Z5TBL7          IPNMVVT--DGSQNRSSPFTAYAMHQVEQRDNFMILTGQRVTEIVWKE-------GDDMI  305
A0A074W657          IPNMVQP--GESQNRSSPYTAYILGEPEKRGNLVILTGHRAVEIRWRNNCTSLESDRILT  281
A0A1Y2DT19          IPNMVQP--DESETRASPYASWIKGTADTRPNLVILLGHRVVKLDWSSTE----EGADLV  289

A0A0D1DW37          TPNSI----GVNGQRTSAASAYYTPVQ-NRDNLTILTGTMAKNLLWDAAT----SSNLLR  302
A0A081CFT4          TPNSI----GTNARRTSSASAYYTPVQ-GRENLTVLTGTRARNLLWGAQS----ASG-LG  293
A0A177U6A2          TPNSMLP--GSGNLRSSSATAYLTPIEQKRSNLVVLTTWRGWKTTWSSTA----RT--PT  316
A0A177UVE5          FPNTFLP--GPGKLRSSSAIAYLSPIEFSRSNLVVLTNFRGWKLIWDPTK-----N--AT  305

ODH                 TPLSINW--HDDDHRSSSIEAYYTPVENNRQGWTLLIDHMATKVLFDG-T----NA-PLT  260
A0A1B9I658          HPNTISW--QDSDHRSSSATAYYSPVSE-RSNLAILLQHQATKIQFDGN----------K  275
PBK65148            TPLTLNW--HDDDHRSSSVEAYLSPVEGIRTNWLTLAEHQVTKINWANAG----SI-PLT  283
A0A067ST11          TPLTINW--HDSDHRSSSAMAYLTPVESVRTNWLTLVTHQVTKINWNT-G----GI-PLT  285

A0A0L1HZM0          WPMTLNE---TGPTREDGARAFYYPVA-SRPNLHVFPNSMATRIIWDDSN---SAAAGVL  272
A0A1L9P605          WPQTLDR---EANVREHSARAYYYPVR-DRSNLVIF-RGRVDRVSWAETN---ES--KVL  265
A0A1W2TMR5          WPQTLDR---DRNIRFDAARAYYYDIE-DRPNLTLI-KGTVKKLTWASCT---ASGQDAV  276
A0A024S2J7          WPQTLDP---VKNVRWDAATAFYWPVQ-DRPNLALL-NGTVSRILWKNDE---AD--VPE  272

GDH_A2QM15          YPSTIDY---TAYVREDAARAYYWPIA-SRPNLHLMLDTFVNRLVWKNGG---SQ-GNAT  274
GDH_4YNU_B8MX95     YPSTLDV---DLNVREDAARAYYFPYD-DRKNLHLLENTTANRLFWKNGS---A--EEAI  271
GDH_G8E4B4          YPKTLNQ---DLNIRWDAARAYYFPYE-NRTNLKVVLNTTAKKLTWASAT---NG-TDAT  276
GDH_G3YD20          HPDTLDR---EMNVREDAARAYYWPYE-ARSNLKIISNTRANKVIWANTT---Q--GEAV  270
                                  *      :       * .   .      .


GOX_A0A1V6T4B5      AVGVNFGTNK--AVNFNVYAKQEVLLAAGSAISPLILEYSGIGIKSVLDKAGVKQLLELP  343
GOX_1CF3_P13006     AVGVEFGTHK--GNTHNVYAKHEVLLAAGSAVSPTILEYSGIGMKSILEPLGIDTVVDLP  343
GOX_1GPE_Q9URJ8     AVGVNFGTNK--AVNFDVFAKHEVLLAAGSAISPLILEYSGIGLKSVLDQANVTQLLDLP  343
GOX_Q92452          AVGVNFGTNK--AVNFDVFAKHEVLLAAGSAISPLILEYSGIGLKSVLDQANVTQLLDLP  343

A0A139IH18          ARGVEISNVD--NK-TVVRARHEVILSAGVYRTPGILEYSGIGNPKILKAHKIDTKIELP  312
M2ZEM9              AHGVEISNVD--NK-TVIHARKEVILSAGVYRTPGILEYSGIGNPKILKSHKIDTKVDLP  312
K2RH81              AAGVEVATAD--GTTAVLNATQEVILAAGAYRSASILEYSGVGNSQILQGLGIATKVYLP  320
R1EB07              AAGVEVASAD--GSTATLNATAEVIVAAGAYRSASILEYSGVGNSQILQSLGIDTKVYLP  324

A0A139HTB9          ADGVHFQSS-KNSPVQFVKANREVLLAAGSLQSPQILELSGVGDSDVLGAAGVELKHELK  356
A0A1Z5TBL7          AERVHFQGC-RDCDISFVKADREVLLSAGSLQSPQILELSGVGDPDVLEAAGVPLKKAAF  364
A0A074W657          ADGVYYQAD-RHSERLFAKATREVLLAAGSMQSPQLLELSGVGDPAILDKAGVPVRKALM  340
A0A1Y2DT19          ASGIQFQQS-RSSPILTAHASRDVILAAGSMQSPQLLELSGVGDATVLANAGIPLVKSVP  348

A0A0D1DW37          SSGVVVQQG-RNGNQIRLVANKEVILAAGALNTPVLLQRSGVGAKTDLNSIGVDQRIELA  361
A0A081CFT4          SSGVVVQQS-HDGNLVTINAKREVVLAAGALNTPTLLQRSGVGAKADLDSIGVDQKLALD  352
A0A177U6A2          ATGVIIQQK-NGGPTYNVKASREIIVAAGAIRSPVFLEHSGIGDANILRNINVPVKVDLP  375
A0A177UVE5          ATGVVIQQS-PGGPTYNVSAKREVIVAAGSIKSPIFLEHSGVGNATVLSKLRVPLKVNLP  364

ODH                 AVGIEFGASDATGNRYKAFARKEVILAAGAIQTPALLQLSGIGDSDVLGPLGISTLSDLK  320
A0A1B9I658          ATGVEYGTSD--GQRYTVNAGKEVIISAGAIQTPALLQLSGVGDPALLNGLGINVVANVS  333
PBK65148            ASGVEFAPTSGGSTRYTATANREVIIACGSIQSPALLQLSGVGDSDVLGPLGIDTHIDLK  343
A0A067ST11          ASGVEFAPASGGSTRYTASARREVILAAGAIQTPALLQLSGIGDSAILGPLGITTMIDLK  345

A0A0L1HZM0          ASGVEIVTAK--DVTETIHASKEVVVAAGAIRSPAFLEHSGVGNPAVLEPLGIETVNPLH  330
A0A1L9P605          AEGVEYTTED--GKAKILYAEKEVIVSAGAVRTPAILELSGVGNPELLKQLDIPVKVPLS  323
A0A1W2TMR5          ADGVEYVTPD--GGLIKVTAAKEVILSAGALRSPLILESSGVGNPSLLSKLGIKPKIDLP  334
A0A024S2J7          ASGVEYLTPD--GNIKTVNARREVILSAGALRTPLILELSGIGNPSILNGLGIETVVNSP  330

GDH_A2QM15          AAGVEITSSN--GTISVIGASQEVIISAGSLKSPGILELSGIGNRDILERYNISVRVDLP  332
GDH_4YNU_B8MX95     ADGVEITSAD--GKVTRVHAKKEVIISAGALRSPLILELSGVGNPTILKKNNITPRVDLP  329
GDH_G8E4B4          ASGVEITAAD--GTTSVVTANKEVIISAGALVSPLLLELSGVGNPAVLSQYGIETVVELP  334
GDH_G3YD20          AVGIEVTNAY--GTET-IYADKEVILSAGALRSPAILELSGIGNPDVLNKHNIPVKVNIT  327
                    :  :               *  :::::.*   :  :*: **:*    *    :
```

```
GOX_A0A1V6T4B5      -VGLNMQDQTTTTVRSRANN-----------APGQGQAAYFANFTEVLGDHAAQGIELLD  391
GOX_1CF3_P13006     -VGLNLQDQTTATVRSRITS----------AGAGQGQAAWFATFNETFGDYSEKAHELLN  392
GOX_1GPE_Q9URJ8     -VGINMQDQTTTTVSSRASS----------AGAGQGQAVFFANFTETFGDYAPQARDLLN  392
GOX_Q92452          -VGINMQDQTTTTVSSRASA----------AGAGQGQAVFFANFTETFGDYAPQARELLN  392

   A0A139IH18       GVGENLQDQAQGNIFFKKDENSTITFPATVGEEITTNYLIHATYEDILGDEAPAVQRRVN  372
   M2ZEM9           GVGENLQDQAQGNIFFNKNSNASITFPDTVGEEIITNYLIHATYEDILGNEAASMQQRVN  372
   K2RH81           GVGEHLMDQANNILTFEPAPEA--------GFTGTTAYVAYASVADVFGDEAAAVAEEVR  372
   R1EB07           GVGEHLMDQANNILTFEPAADA--------GFTGTTAYVAYANVTDVFGPDAPTVAEEVR  376

     A0A139HTB9     GVGAHMQEQTKNTLTYQARPGVNY------NGTGPPSSIVFPDAAQLLGENATTWYSTVK  410
     A0A1Z5TBL7     GVGKHMQEQTKNSVVQNAQ-EMEY------DGSGPPSGIAFPNVHQLLKDNSSATYDYVM  417
     A0A074W657     GVGKNLQEQTKNTIIYTPK-SDDF------EGSGPPSAIAFPNVHQLLGNKSSAVHKETL  393
     A0A1Y2DT19     GVGKNLQEQTKSSLYYTPI-STAW------NGTGPSTAIAFPNAWQLLRSNASAVYNATI  401

     A0A0D1DW37     GVGKNLQDQTMTTIGSR--ANVNY------AGGGPSATIAMPNIQQIMS-NSTAVRSYIT  412
     A0A081CFT4     GVGKNLQDQTMTTLGSR--ANVNY------SGGGPSAAIGMPNVNQVFS-NATAVRSYIS  403
     A0A177U6A2     GVGKNLQEQTMSVFGASRKNGVSF------GGVGPSNLIAQPAAGQLFS-NATAVRSYIE  428
     A0A177UVE5     GVGANLQEQTNGVLGIPN-GTIPY------GGVGPGNVVAFGSVAQLMPNNVTAVRQAIE  417

  ODH               TVGKNLQEQTQNAIGAK-GNGFDP------DGHGPTDAIAFPNIYQVFGSQATSAVQTIQ  373
  A0A1B9I658        GVGKNLQEQTMNSVGWTPVDGFDF------DGRGPSDCIAYPDLLGISSSSNNDIASTIS  387
  PBK65148          TVGRNLQEQTMNSLGAD-GN-VDY------GGSGPSDAIAYPNIYQVFGDEAAASVSKIQ  395
  A0A067ST11        TVGKNLQEQAMSTHGAG-GNGFDE------GGRGPTDVIAYPNLYQIFGSNANATVSRIQ  398

A0A0L1HZM0          SIGANLPDQPQNGMVFSSSTNWTG----------YPTFVSYLTASDLFGEELAGLAEEVR  380
A0A1L9P605          SVGENAIDQPNTFISYSSTKNFTG----------IAPYVTYMTASDVFGPETQRIADEIS  373
A0A1W2TMR5          GVGEYLQEQPNTALVYSGTQNVSG----------FAPYATFSTAGDLFGDETPAIKASTS  384
A0A024S2J7          GVGENLIDQSNVALTYSTKESFPG----------YAPYATFVNATSLFGDGVEALAASTK  380

GDH_A2QM15          TVGENLQDQTNAGLGASTTPGLTG-----------TKTVVYPNVYDVFGNDTLAVAQSVR  381
GDH_4YNU_B8MX95     TVGENLQDQFNNGMAGEGYGVLAG-----------ASTVTYPSISDVFGNETDSIVASLR  378
GDH_G8E4B4          TVGENLQDQINNELIYSPPTNFTSTY-----DSGVGAFVAYPSASHVFGTNESSASEELK  389
GDH_G3YD20          TVGENLQDQTNNALSWEGVDTLTG----------LATFSVLPSVNQLYGDNVTALASYVK  377
                      :*     :*


GOX_A0A1V6T4B5      TKLDQWAEETVARGG--FHNVTALKIQYENYRNWLLDE--DV-AFAELFFDTE-------  439
GOX_1CF3_P13006     TKLEQWAEEAVARGG--FHNTTALLIQYENYRDWIVNH--NV-AYSELFLDTA-------  440
GOX_1GPE_Q9URJ8     TKLDQWAEETVARGG--FHNVTALKVQYENYRNWLLDE--DV-AFAELFMDTE-------  440
GOX_Q92452          TKLDQWAEETVARGG--FHNVTALKVQYENYRNWLLDE--DV-AFAELFMDTE-------  440

   A0A139IH18       ASLGDYATMISNRINNS-LSADQILNSLKVQYEAIFDT--PV-PVVELFSGQAI----NN  424
   M2ZEM9           ASLGDYATTISNRINNS-LSSDQILNSLQVQYDAIFNT--PV-PVVEIFSGQAV----NN  424
   K2RH81           AALPDYAATIAAQNNNA-TSAEELLPLLEIQYTSIFET--GI-TAFELLKGLEF---QP-  424
   R1EB07           AALPDYAATIASQNNNA-TSAEDLLPLLEIQHASIFDA--QI-TAFELLKGLEF---QP-  428

     A0A139HTB9     SGLQDYANSLEERSL--VANAKATHHILQAQLDNIFNA--TDVAASEIFFTINT----TH  462
     A0A1Z5TBL7     ETLPQYCQELQEKGL--VVNATATHTILEAQVNNLFKQ--NA-AASEVFFTVSP----GT  468
     A0A074W657     ANLASYAADLEAQGL--VANATATHEILRLQVANLFDQ--SE-AAAEIFFTLAATDSAP  448
     A0A1Y2DT19     ASLPAYAAQLAASGS--VVNATATAQIMRLQIESLFAN--NE-PAAEVFWTIDT----AG  452

     A0A0D1DW37     SNLDGWANQLLSQGH--VASKEGVLAQWRSAISLIFDQ--KA-PVVELFFDTGF----PA  463
     A0A081CFT4     SNLNAWADTLVSAGH--VASKSGLLAQWTSAVSLIFDQ--AA-PIVEMFFDTGY----PA  454
     A0A177U6A2     SNYQNWAQAAVSGGG--AVNTDGLIAQWRLQTEVLFTD--NV-GAVEMFVDSGY----PN  479
     A0A177UVE5     SKYTTWARDAVVAGA--AVNSAGLIAQWKLAVSALFDY--NV-GATEFLFTTGF----PT  468

  ODH               SSLSAWAKTQAAAGA---LSADALNTIYQTQADLIINH--NA-PVVELFFDSGF----PD  423
  A0A1B9I658        DNIAQYAQDAYDAGA--VASVEAANTIFGIQQDLMVNK--NS-GLVEAFFDSGF----PN  438
  PBK65148          SSLSSWASSQAG-SA---LSADALEQIYNIQADLIINQ--NA-PIVELFFDSGF----PA  444
  A0A067ST11        SSIASWAASQAD-SG---LSANALQQIFQVQADLIINN--NA-PVMELFLDIGY----PD  447

A0A0L1HZM0          ANLSAYAATIVEDYANGTISVETQEHLLSHQVDLIFDSESKV-PLAELLWAPT------G  433
A0A1L9P605          SEIKSWAEQVSADSPGQGFPASAIEHQYRVQHDLIFNQ--DT-AITEILTTSL------G  424
A0A1W2TMR5          AQIPGWAEKVVAASNGA-VKAANVEKLFRIQHDLIFEK--NV-TIGETLTAIF-----GG  435
A0A024S2J7          KSLPIWAQQIAAQTNGV-ISARAIEHRLQVQHDLIFKK--GV-TIAEILSSAS------G  430

GDH_A2QM15          RQLKQWANETAQVSSGT-MKAEDLEALFQLQYDLIFKD--KI-TIAEILYYPG-----ST  432
GDH_4YNU_B8MX95     SQLSDYAAATVKVSNGH-MKQEDLERLYQLQFDLIVKD--KV-PIAEILFHPG-----GG  429
GDH_G8E4B4          SQLTAYADTVAIANGNV-TKASDLLDFFQLQYDLIFKD--QV-PFAEVLIYIA-----KG  440
GDH_G3YD20          SQLASYAKTVASASNGA-VKEANLVEAFERQYDLIFNS--QV-PYTEVVFAPS-----G-  427
                            :.                        :.         * .
```

Substrate-binding loop

```
|GOX_A0A1V6T4B5     GKINFDIWNLIPFTRGSVHILSSDPYLW----QYANDPKFFMNELDLLGQAAATKLGREL  495
|GOX_1CF3_P13006    GVASFDVWDLLPFTRGYVHILDKDPYLH----HFAYDPQYFLNELDLLGQAAATQLARNI  496
|GOX_1GPE_Q9URJ8    GKINFDLWDLIPFTRGSVHILSSDPYLW----QFANDPKFFLNEFDLLGQAAASKLARDL  496
|GOX_Q92452         GKINFDLWDLIPFTRGSVHILSSDPYLW----QFANDPKFFLNEFDLLGQAAASKLARDL  496

  |A0A139IH18       TTVNMEFWPLIPLSRGSCHIAGSNADLAAQEP--FIDVNWFMLEWDWDIYIASGRFVRKL  482
  |M2ZEM9           NVVNMEFWPLIPLSRGSCHIAGANADLAAEAP--FIDVNWFMLEWDWEIYIAAGRFVRKL  482
  |K2RH81           TRLECEVWSTLPFSRGNVHITTARPPTDGSTPALAINNNYFQLDYDNRAQIAAARFVRNL  484
  |R1EB07           TRLDCEVWSTLPFSRGNVHITTAAAPTDGSTPALAINNNFFQLDYDTRASVAAARFVRRL  488

    |A0A139HTB9     NLIGIDNWNLIVLSRGSAHIRTNSTWDN-----PVVNPSYFDHPLDQEFQLATQKLSRQI  517
    |A0A1Z5TBL7     GQLGIDFWNLIVLARGTAHISSNSSWDH-----PIVEASYFGHPLDMMIQRETCKQSREV  523
    |A0A074W657     ATVGADLWNLIVLARGTIHITSNNSFDL-----PEIEPSYFGHPLDLQLQTLATQQAAQV  503
    |A0A1Y2DT19     QTVGADIWNLIPLARGTVHVQSDNSWDQ-----PAIDAAYFGHELDTTLQVLATRQARDV  507

    |A0A0D1DW37     NSYGIDIWTLLPFSRGSIRATSQNPYDG-----ARIDPNYFGLPIDMDMQVASLRASRRV  518
    |A0A081CFT4     NSYGIDLWTLLPFSRGSVRATSGNPFDA-----PRVDPNYFGLPIDMDMQVQSLRAARRV  509
    |A0A177U6A2     NGFGVEMWPLLPYSRGSVHTSSASTFAK-----TIVDPRYFSVPFDMDMQVAGCRGVRRV  534
    |A0A177UVE5     NDYGIELWPLLPFSRGYVHAVSADAFSN-----ATVNPRYFSVPFDMDLQVASSRAARRI  523

   |ODH             -DVGIVMWPLLPFSRGNVTITSNNPFAK-----PSVNVNYFSVDFDLTMHIAGARLSRKL  477
   |A0A1B9I658      GGLGIDLWQLLPFSRGTVKITSTDPFNY-----PSIDPRYFSADVDLKIQIAGLRMARKI  493
   |PBK65148        -TLGIDQWQLLPFSRGTVQISTTDPFTQ-----PAVNVNYFSVDWDLDVQIASARLSRTI  498
   |A0A067ST11      -TLGMLAWNLLPFSRGNVKITTTDPFTQ-----PQVTVNYYSVDFDLDVQVAGARLARKI  501

|A0A0L1HZM0         NQIIAQLWNLLPLSRGSIHINSTDATLP-----PRIDPAFLQLPIDRYVQAAAAIRVRKY  488
|A0A1L9P605         PLVGTAFWISLPFSRGSVHIHSSDPGVY-----PSLDPNYFAAEWDLVFQRRIAQLIFRY  479
|A0A1W2TMR5         EFLVSAMWLLLPFSWGSVHLTSVDAINA-----PAIDPKYFLVDFDLDVQIGLGRTAQKF  490
|A0A024S2J7         TTAISAYWDTLPFSRGSVHLSSANDINT-----PAINPRFLSVDFDLAVQVAVGRLATKF  485

|GDH_A2QM15         SSISAQYWALMPFARGHVHIASADPTAK-----PVINPNYYKFDWDLTSQIAVAKYVRKT  487
|GDH_4YNU_B8MX95    NAVSSEFWGLLPFARGNIHISSNDPTAP-----AAINPNYFMFEWDGKSQAGIAKYIRKI  484
|GDH_G8E4B4         -SWGAEYWGLLPFSRGSIHISQANSTAG-----ALINPNYFMLDYDVELQVATAKFIRSV  494
|GDH_G3YD20         NSFAVEYWPLLPFSRGSVHIQSANASDY-----PAINPNYFMFDQDAEAQVTVAQYIRKA  482
                           *  :  : *                            :     *


|GOX_A0A1V6T4B5     SSAGEMKKY-YAGETIPGDNL--------PQDA--TVEQWEDYVM-------MNFRPNWH  537
|GOX_1CF3_P13006    SNSGAMQTY-FAGETIPGDNL--------AYDA--DLSAWTEYIP-------YHFRPNYH  538
|GOX_1GPE_Q9URJ8    TSQGAMKEY-FAGETLPGYNL--------VQNA--TLSQWSDYVL-------QNFRPNWH  538
|GOX_Q92452         TSQGAMKEY-FAGETLPGYNL--------VENA--TLSQWSDYVL-------QNFRPNWH  538

  |A0A139IH18       FATPPLANF-TTEETQPGLDNL-------PIDA--SDDEWRDYWS-------DNFRAGWH  525
  |M2ZEM9           FATQPLANL-TTEETQPGYKNL-------ALDA--SDDEWKDYWS-------DNFRAGWH  525
  |K2RH81           YALPPLNGSSVLDEIAPGLATV-------PPDA--TDDEWEVWLK-------GQFRSAWH  528
  |R1EB07           YGLAPLNGSAVLDEIAPGLATV-------PPDA--GDDEWEVWIK-------GQFRSAWH  532

    |A0A139HTB9     FNTEPLASYV-VEELEPGLDRV-------PVHA--SDEAWEAWMK-------REFTSVWH  560
    |A0A1Z5TBL7     YQTEPLAQYV-KSELVPGKA-V-------SQEG--TDEEWEQWMK-------ETFTSVWH  565
    |A0A074W657     YSTSPLAEMV-DKNILPATLP-----------V--TYEAWETFVK-------DTFTSVWH  542
    |A0A1Y2DT19     YAAEPLASMV-SGEITPGLERV-------ALEA--DDAAWTEWVK-------SEFTSVWH  550

    |A0A0D1DW37     LQNSNLRSLTYNGETTPGFSLI----PDGPNSG--RYSRWRDWILGTLPNGGSGFAAVSH  572
    |A0A081CFT4     LKNSHLRSLTYNGETTPGFGLI----PDGPNSG--SYAKWRAWILGTLQGGGSGFSAVSH  563
    |A0A177U6A2     YQTSPVSDLFAAGEEIPGFDVSRGGIPDGPKHG--AFARWQKWIS-------EGYSSVAH  585
    |A0A177UVE5     LQGDAFKNISSGPENKPGFKVVP---DDSVNHG--SYATWQAWIS-------KNFGSVAH  571

   |ODH             LGSPPLSSLL-VGETVPGFKTV----PNNGNGG--TDADWKKWIL--KPGNSAGFASVAH  528
   |A0A1B9I658      FQTSPLRSIV-TEENAPGYNEV----PENNDGG--SDEDWSNWIL-------NGFSSVHH  539
   |PBK65148        LSSPPLSDLS-TGEVIPGD-SV----PDNGERG--TEEDWTSWIT-------NGFAAVSH  543
   |A0A067ST11      LTTPPLSSLS-TGETRPGS-AV----PDNASRG--TDAAWKSWIK-------STFDSVAH  546

|A0A0L1HZM0         FATSPLADH-ITGELSPGFETV-------PQDAGWRDASWDAWIK-------QTLGGNSH  533
|A0A1L9P605         WGTDPVRSL-AGQRLQPAVGDL-------PSNA--TDEALDKWIA-------SSFSANQH  522
|A0A1W2TMR5         WNTEPVSDI-VGARVSPTDDDV-------PLNA--TDEQWAGYIS-------DSLTPNHH  533
|A0A024S2J7         WTTAPISAV-IEARVDPNSTIL-------PDNA--TDAQWEDFTR-------SSLISNSH  528

|GDH_A2QM15         FQSAPLANI-IAEETNPGFEAV-------AANG--SEEDWKAWLL-------TQYRSNFH  530
|GDH_4YNU_B8MX95    LRSAPLNKL-IAKETKPGLSEI-------PATA--ADEKWVEWLK-------ANYRSNFH  527
|GDH_G8E4B4         FGTGPFASV-AGTETTPGFDVI-------PADA--DEATWKSWAT-------KEYRSNFH  537
|GDH_G3YD20         LGTAPLNSL-VGEEVSPGLDVL-------PASA--SSATWTKWVK-------ENYRTNYH  525
                         .        .   *                            :           *
```

```
GOX_A0A1V6T4B5     AVSTCSMMSRELGGVVDATAKVYGTQGLRVIDGSIPPTQVSSHVMTVFYGMALRIAESVL   597
GOX_1CF3_P13006    GVGTCSMMPKEMGGVVDNAARVYGVQGLRVIDGSIPPTQMSSHVMTVFYAMALKISDAIL   598
GOX_1GPE_Q9URJ8    AVSSCSMMSRELGGVVDATAKVYGTQGLRVIDGSIPPTQVSSHVMTIFYGMALKVADAIL   598
GOX_Q92452         AVSSCSMMSRELGGVVDATAKVYGTQGLRVIDGSIPPTQVSSHVMTIFYGMALKVADAIL   598

  A0A139IH18       GVGTAAMLPRKWGGVVNGDLKVYGTSNVRVVDASAVPFNMGGHPTSTMYALAERAAHLIL   585
  M2ZEM9           GIGTAAMLPRKWGGVVDGNLKVYGTSNVRVCDASAVPFNMGGHPTSTMYALAERAAHLIL   585
  K2RH81           PVGTTSMLPRQNGGVVDAELKVYETANVRIVDAGVFPFQINGHPSATVYAVAERAADIIK   588
  R1EB07           PVGTTSMLPRAAGGVVDPQLRVYETANVRVVDAGVMPFQVNGHPSATVYAVAERAADMIK   592

    A0A139HTB9     YVATCAMMKEEFGGVVDSKLKVYGIENVRVVDASVLPIQLSAHLSSSLYGIAEKGAQMVR   620
    A0A1Z5TBL7     YIATLAMMKEEYGGVVDNRLKIYGIENVRAIDASVLPIQLSAHLSSSLYGIAEKAAAMIK   625
    A0A074W657     PIATLSMMKEEFGGVVDNKLKVYGLSNVRVVDASVLPVQLSAHLSSSLYGIAEKAAVMIR   602
    A0A1Y2DT19     PVATLSRMAEQDGGVVDESFRVYGVQNVRVVDASVLPVQLSAHLSSSLYGIALKAAGIIR   610

    A0A0D1DW37     QLGTAAMGSRSLGAVVDAKFKVYGTSNVRVVDASVLPVQISAHLSSTLYGVAEKAADTIL   632
    A0A081CFT4     QLGTAAMGSKQNGGVVDGNFKVYGTTNVRVVDASVVPVQLSAHLSASLYGVAEKAADAIL   623
    A0A177U6A2     PVGTCALLPRQMGGVVDASFKVYGTANVRVVDASTLPQLISAHLSSTLYGIAERAADQIS   645
    A0A177UVE5     PIATCSLAPRAMGGVVDPNFLVYGTTNVRVVDASTLPMQISAHLSSTLYGIAERAADSIK   631

   ODH             PIGTAAMMKRSLGGVVDAQLKVYDTTNLRVVDASMMPLQISAHLSSTLYGVAEKAADLIK   588
   A0A1B9I658      PIATCSMMSEELGGVVGSDLKVYNTENLRIVDASVLPIQFSAHLSATLYGLAENAADM--   597
   PBK65148        PIGTLAMMKRSLGGVVDSQLKVYDTSNVRVVDASIVPIHISAHLSAGLYGVAEKAADLIK   603
   A0A067ST11      PIGTAAMMKRSLGGVVDSQLKVYGTTNVRVVDASIMPLQISAHLSSTLYGVAEKAADLIK   606

A0A0L1HZM0         PLSTCAMMQKDLGGVVDTEGKVYGTQNVRVVDASIFPTQISGHLSASVYAVAGRIAAVMM   593
A0A1L9P605         LVGTASMLPMEFGGVVDTNLVVYGTENVRVVDASVLPGQNSGHLTSLIYAVAERAADIIK   582
A0A1W2TMR5         PLGTASMMSRELGGVVDAELKVYGTGNVRVVDASVLPIQFSGHLTATLYAVAERAAEIIA   593
A0A024S2J7         SLGTAAMMKREWGGVVDSQMRVYGTGNVRVVDASVLPMQVSGHLTATLYAVAERAGEMIV   588

GDH_A2QM15         PVGTAAMMPQDKGGVVNDRLTVYGTSNVRVVDASVLPFQVCGHLVSTLYAVAERASDLIK   590
GDH_4YNU_B8MX95    PVGTAAMMPRSIGGVVDNRLRVYGTSNVRVVDASVLPFQVCGHLVSTLYAVAERASDLIK   587
GDH_G8E4B4         PVATAAMLPKEKGGVVDAQLKVYGTTNVRVVDASVLPFQVCGHLVSTLYAVAEKASDLIK   597
GDH_G3YD20         PVGTTSMLPREKGGVVSPELKVYGTKNVRVVDASVLPFQLCGHLTSTLYAVAERASDLIK   585
                    :.: :      *.**.    :*   .:*  *..  *    .*  : .*.:* . .


GOX_A0A1V6T4B5     EDYAKKA-------------------------------------------   604
GOX_1CF3_P13006    EDYASMQ-------------------------------------------   605
GOX_1GPE_Q9URJ8    DDYAKSA-------------------------------------------   605
GOX_Q92452         DDYAKSA-------------------------------------------   605

  A0A139IH18       TGEKG---------------------------------------------   590
  M2ZEM9           TGEKG---------------------------------------------   590
  K2RH81           QSFSAEIEIFNWHLTISTQNHQPLKVKPPVDISKPRNEELFSQTCLAT   636
  R1EB07           ESFAGGGVGASGGTEGSG-TGTPGA-AARMGRVKGRVVRL--------   630

    A0A139HTB9     LDWGGE--------------------------------------------   626
    A0A1Z5TBL7     EDQKGFREQPWAHQH-----------------------------------   640
    A0A074W657     EDQI----------------------------------------------   606
    A0A1Y2DT19     EDQ-----------------------------------------------   613

    A0A0D1DW37     AR------------------------------------------------   634
    A0A081CFT4     AK------------------------------------------------   625
    A0A177U6A2     ASQ-----------------------------------------------   648
    A0A177UVE5     AAQT----------------------------------------------   635

   ODH             AAQ-----------------------------------------------   591
   A0A1B9I658      --------------------------------------------------   597
   PBK65148        ASY-----------------------------------------------   606
   A0A067ST11      ASY-----------------------------------------------   609

A0A0L1HZM0         KAVTMK--------------------------------------------   599
A0A1L9P605         ETL-----------------------------------------------   585
A0A1W2TMR5         GAV-----------------------------------------------   596
A0A024S2J7         GDRSGL--------------------------------------------   594

GDH_A2QM15         ADSALF--------------------------------------------   596
GDH_4YNU_B8MX95    EDAKSA--------------------------------------------   593
GDH_G8E4B4         AAA-----------------------------------------------   600
GDH_G3YD20         ESY-----------------------------------------------   588
```

Figure S7. Close-up view of the active site flavin. Protein and FAD carbon atoms are shown in wheat and yellow respectively, O atoms in red, N atoms in blue. **a** Interactions between the isoalloxazine ring and the protein. The isoalloxazine ring is rigidly fixed through a dense network of hydrogen bonds. Most contacts involve the protein backbone except for Ser573, which stabilizes the cofactor also with its side chain. Ser573 is modelled in double conformation: one conformer interacts with the flavin system, the other interacts with Asn97 side chain. Asn97 is located on the *si*-face of the isoalloxazine ring, while the two catalytic histidines, His528 and His571, lie on the *re*-face: the orientation of their imidazole ring is due to hydrogen bonding with Gln329 and Glu414 respectively. **b** Residues facing the active site. Except for Gln331, most of the residues directly facing the active site possess aromatic side chains: Tyr64, Phe416 and Trp430. Water molecules are depicted as red spheres. Hydrogen bonds (distance < 3.2 Å) are represented with a dotted line.

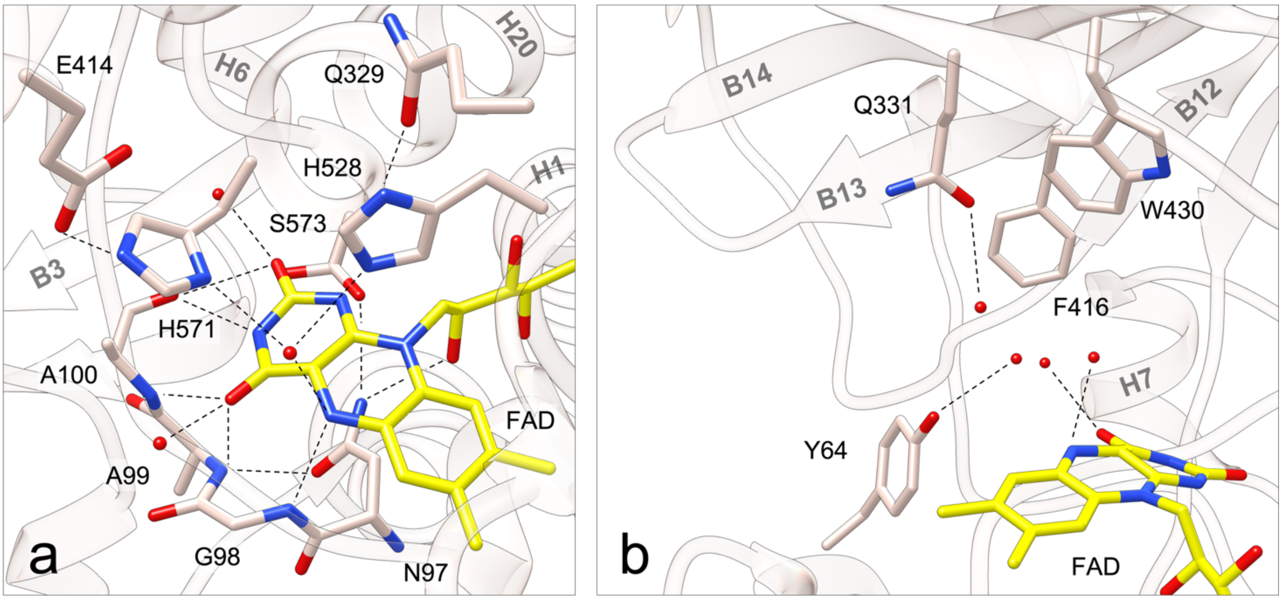

Figure S8. Details of the two anomeric forms of glucose (GLC) and laminaribiose (G3G) bound to ODH active site, **a** Top view (perpendicular to the isoalloxazine plane) and **b** side view of β-G3G in ODH-G3G; **c** top view and **d** side view of α-G3G in ODH-G3G; the protein is shown in green, the FAD cofactor in yellow, G3G in pink (β anomer) or magenta (α anomer). **e** Top view and **f** side view of β-GLC in ODH-GLC; **g** top view and **h** side view of β-GLC in ODH-GLC; the protein is shown in light blue, the FAD cofactor in yellow, GLC in orange (β anomer) or gold (α anomer); O atoms are in red, N atoms in blue. Water molecules are depicted as red spheres. Hydrogen bonds (distance < 3.2 Å) are represented with dotted lines.

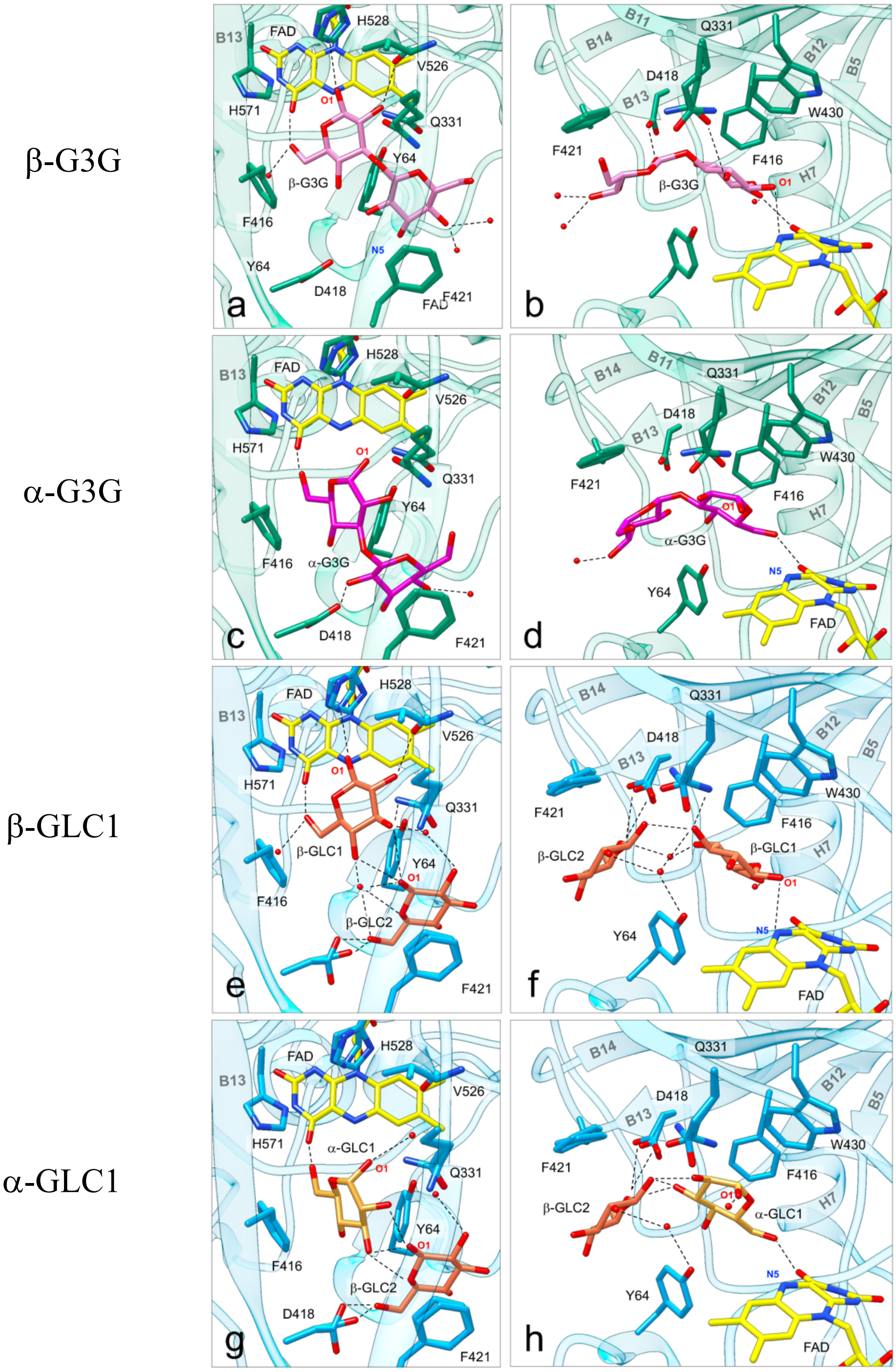

Figure S9. PDBe Motif analysis of protein-ligand interactions in ODH-G3G, ODH-GLC and *Af*GDH-LGC. Comparison of the simplified PDBe outputs for: i) (upper panel) β- and α-LMB reducing end pyranose in ODH-G3G (pdb: 6XUV) and gluconoactone (LGC) in *Af*GDH-LGC (4YNU); ii) (lower panel) β- and α-LMB non-reducing end pyranose in ODH-G3G (pdb: 6XUV) and GLC2 in ODH-GLC (pdb: 6XUU). As for the reducing end of β- and α-G3G, similar results were obtained for α- and β-GLC1 in ODH-GLC (not shown). Colors represent different bond types. Atom pairs, distances and eventually angles are reported. No van-der-waals interactions have been shown, apart from two crucial ones between Tyr53 hydroxyl and LGC in *Af*GDH-LGC (for more details see Additional file 2, Section 4).

Ligand bond colours:	Phylogenetically relevant H bonds (side chains)	H bonds (others)	electrostatic	van-der-waals	atom-plane	plane-plane

- ligand-protein interactions for βLMB reducing end (ODH-LMB, 6XUV):

Atom	Bond	Neighbour
O1	3.04Å	NE2.HIS 528A
	2.98Å	N5.FAD 601A
O2	2.80Å	O.VAL 526A
	3.14Å	OE1.GLN 331A
O3	2.99Å	OE1.GLN 331A
O6	2.78Å	O4.FAD 601A
C3	3.38Å	OH.TYR 64A
C6	3.35Å/22918°	CD1,CD2,CE1,CE2,CG,CZ.PHE 416A
C1,C2,C3,C4,C5,O5	4.25Å/27°	CD2,CE2,CE3,CH2,CZ2,CZ3.TRP 430A

- ligand-protein interactions for αLMB reducing end (ODH-LMB, 6XUV):

Atom	Bond	Neighbour
O6	2.48Å	O4.FAD 601A
C4	3.44Å/22918°	CD1,CD2,CE1,CE2,CG,CZ.PHE 416A
C1,C2,C3,C4,C5,O5	4.32Å/30°	CD1,CD2,CE1,CE2,CG,CZ.PHE 416A

- ligand-protein interactions for gluconoactone (LGC) (*Af*GDH-LGC, 4YNU):

Atom	Bond	Neighbour
O1	2.79Å	NE2.HIS 505A
	2.57Å	ND1.HIS 548A
	3.07Å	N5.FAD 601A
O2	2.66Å	O.ASN 503A
O3	2.78Å	OD1.ASN 503A
	2.83Å	NH1.ARG 501A
O4	2.60Å	OE1.GLU 413A
	2.89Å	NH2.ARG 501A
	2.71Å	OH.TYR 53A
C3	3.46Å	OH.TYR 53A
C1,C2,C3,C4,C5,O5	4.05Å/37°	CD2,CE2,CE3,CH2,CZ2,CZ3.TRP 415A
	3.98Å/16°	C10,C2,C4,C4X,C5X,C6,C7,C7M,C8,C8M,C9,C9A,N1,N10,N3,N5,O2,O4.FAD 601A

- ligand-protein interactions for βLMB non-reducing end (ODH-LMB, 6XUV):

Atom	Bond	Neighbour
C1,C2,C3,C4,C5,O5	4.09Å/1°	CD1,CD2,CE1,CE2,CG,CZ.PHE 421A

- ligand-protein interactions for αLMB non-reducing end (ODH-LMB, 6XUV):

Atom	Bond	Neighbour
O2	3.10Å	OD2.ASP 418A
C1,C2,C3,C4,C5,O5	4.02Å/23°	CD1,CD2,CE1,CE2,CG,CZ.PHE 421A

- ligand-protein interactions for GLC2 (ODH-GLC, 6XUU):

Atom	Bond	Neighbour
O6	3.10Å	OD2.ASP 418A
C1,C2,C3,C4,C5,O5	4.28Å/26°	CD1,CD2,CE1,CE2,CG,CZ.PHE 421A

Figure S10. Surface representation of ODH substrate-binding cavity in ODH-GLC (6XUU). Four GLC molecules bind to this region, numbered according to their proximity to the active site FAD. They seem to be caught in the diffusion process from the bulk to the flavin system, with GLC4 and 3 binding to the funnel-shaped opening to the solvent of ODH active site, while GLC2 and 1 bind close to the FAD cofactor within a V-shaped tunnel. ODH surface is shown in light blue, GLC molecules in orange (C atoms) and red (O atoms) sticks.

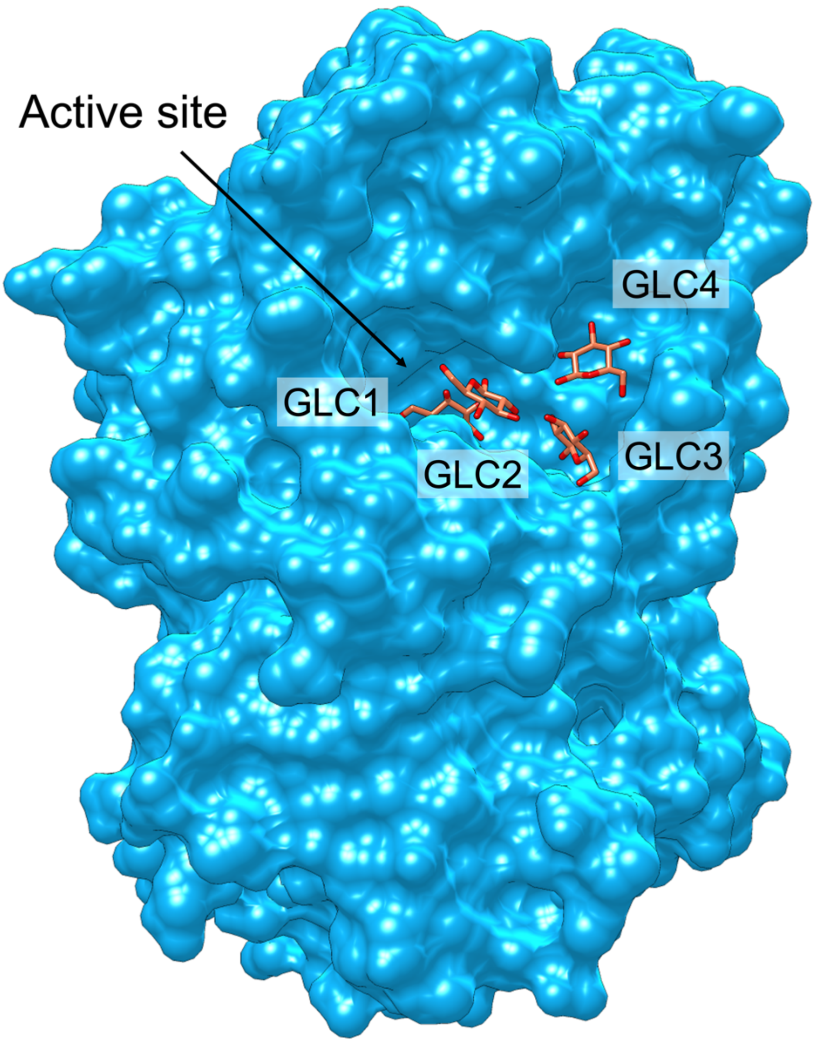

Figure S11. External sugar-binding sites (SBSs) in ODH-GLC (6XUU). All three sites are located on the convex face of ODH, whose surface is represented in the center and colored in light blue. SBSs are numbered according to their proximity to the active site entrance, located on the concave face of the protein (not visible). The three insets show the detail of ODH-ligand interactions. GLC molecules and the surrounding protein atoms are represented as orange and light blue sticks respectively (C atoms); O atoms are in red, N atoms in blue and water molecules are depicted as red spheres. Hydrogen bonds (distance <3 .2 Å) are represented with dotted lines. The $2F_o$-F_c electron density map is also depicted for the GLC molecules and contoured at 1σ.

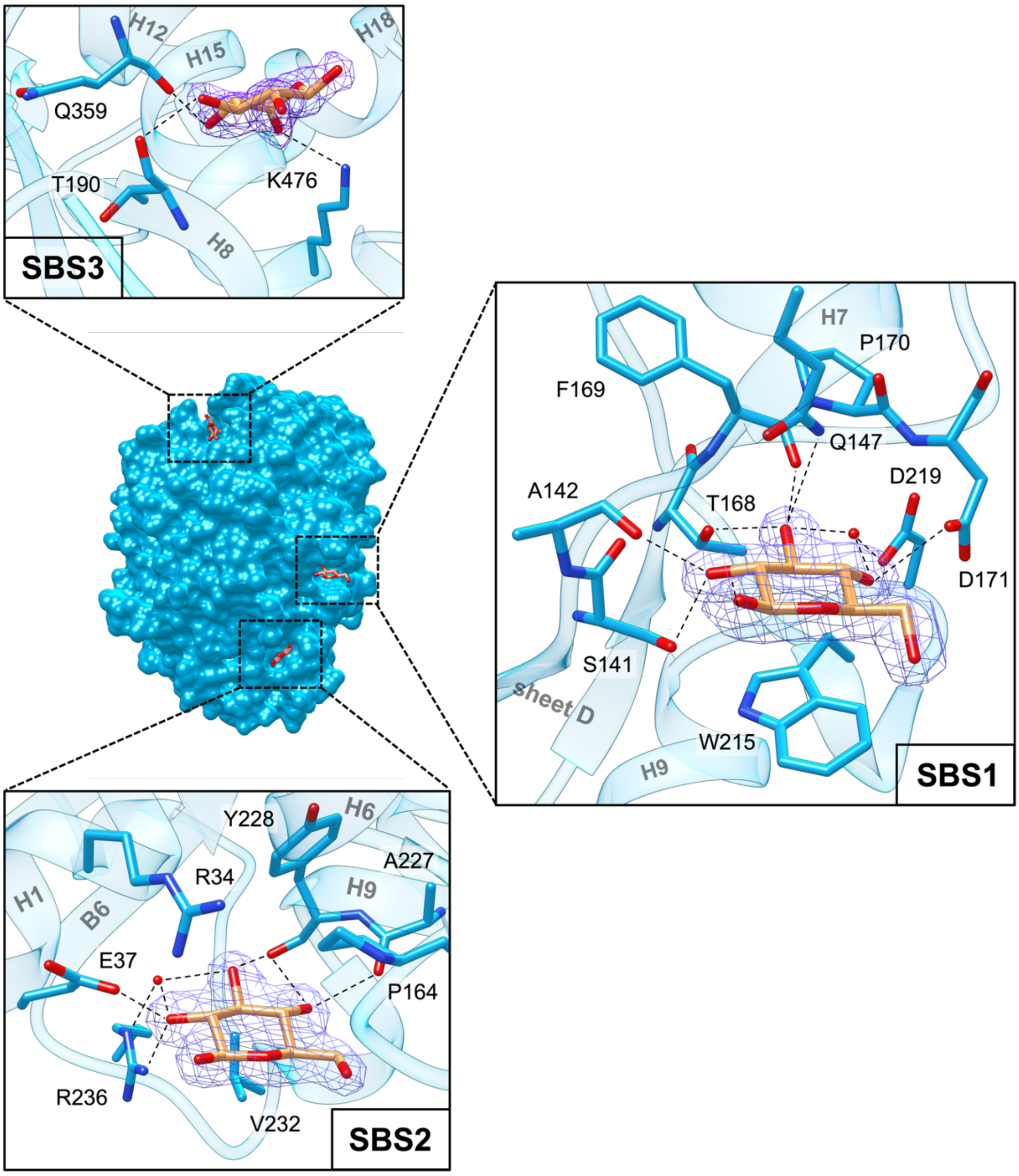

Supplement: Supplementary file 1 — Additional file 1: Supplementary Figures. [file 13068_2021_2003_MOESM1_ESM.pdf]
