# Supplementary material for: Crystal structure and functional characterization of an oligosaccharide dehydrogenase from Pycnoporus cinnabarinus provides insights into fungal breakdown of lignocellulose
Source: Biotechnol Biofuels. 2021 Jul 22;14:161. doi: 10.1186/s13068-021-02003-y (PMC8296622; doi:10.1186/s13068-021-02003-y)
Supplement: Supplementary file 2 — Additional file 2: Key amino acids and interactions: a more detailed analysis. [file 13068_2021_2003_MOESM2_ESM.pdf]

Additional File 2. Key amino acids and interactions: a more detailed analysis

Section 1. Stabilization of substrate-binding loop conformations

The substrate-binding loop (residues 419 to 424, see Additional file 1, Fig. S6) is mostly conserved in the ODH and ODH-like subclades from Basidiomycetes and partially from the Ascomycetes subclades. Ser419 and Asp423 seem to play a role in the stabilization of substrate-binding loop conformations. Ser419 stabilizes the B13 strand in ligand-free and in ligand-bound ODH structures. In fact, on one hand, its side chain binds to Gln178 (hydrogen bond) and Tyr174 (CH-π interaction), on the other hand its peptide amide binds to Thr349 and Asp418 (hydrogen bonds). Tyr174, Gln178, Thr349 and Asp418 are quite well conserved, or replaced by chemical equivalents, in enzymes from Basidiomycetes (subclades ODH and ODH-like), and sometimes in other groups (subclades GOX, GOX-like and ODH-like from Ascomycetes), reflecting the conservation of the substrate-binding loop (see Additional file 1, Fig. S6). Similarly, Asp423 establishes a hydrogen bond with the backbone amide of its C-terminal neighbor (Asp424) only upon ligand binding, possibly stabilizing the ligand-bound conformation of the substrate-binding loop. This residue, unlike Ser419, belongs to the flexible part of the substrate-binding loop and seems poorly conserved.

Section 2. Hydrogen bonds do not play a key role in substrate binding

Few hydrogen bonds are established between G3G or GLC and specific ODH residues, and only with the side chains of Gln331 (β-G3G and β-GLC1) and Asp418 (α-G3G and GLC2). In ODH-G3G and ODH-GLC, both the non-reducing pyranose of α-G3G and GLC2 are at hydrogen bond distance with Asp418, that is conserved in enzymes from Basidiomycota and also involved in the stabilization of the N-terminal hinge of the substrate-binding loop. However, neither of the two amino acids is likely to play a key role in ODH substrate binding. In fact, while Asp418 is only involved in catalytically non-productive (α-G3G) substrate binding, Gln331 is not very much conserved within the ODH and ODH-like subclades, suggesting a mere evolutionary legacy in common with enzymes from other subclades (GDH class-I, GDH class-II and GOX-like), rather than a key role in substrate binding. To further sustain this hypothesis, the equivalent Asn340 does not bind LGC directly in *Af*GDH (subclade GDH class-I, pdb: 4YNU), although it binds two LGC-binding residues (Glu413 and Asn503).

Finally, sequence comparison indicates that the key substrate-binding residues conserved in GOX and GDH class-I (Glu413, Arg501 and Asn503 in *Af*GDH, see Additional file 1, Fig. S9) are not conserved in ODH and related enzymes (see Additional file 1, Fig. S6). As such, another mechanism, not relying on hydrogen bonds, must be required for substrate recognition in ODH (see next section).

Section 3. CH-π interactions involved in substrate binding (sugar β-face)

Substrate-protein interaction analysis of ODH-G3G using the PDBe Motif server [1] shows that both G3G anomers are stabilized by atom-plane and plane-plane bonds with ODH aromatic residues (see Additional file 1, Fig. S9). These bonds, also referred to as sugar-aromatic stacking, rely on CH-π interactions, *i.e.* very weak

hydrogen bonds established between aromatic π-systems (weak H acceptors) and single or multiple C-H groups (weak H donors) of sugar aliphatic heterocycles. In the case of multiple C-H donor groups, because of their cooperative nature, these bonds may give rise to important interactions resulting in sugar-aromatic plane-plane stacking [2]. CH-π bonds are thought to be important for the function of biological macromolecules [3], notably contributing to protein structure integrity [4] and to protein-sugar interactions, such as for sugar recognition, enzyme processivity on polysaccharides and eventually catalysis [5]. A recent analysis of protein-sugar interactions in structures deposited in the PDB, accompanied by quantum chemical calculations, showed that CH-π interactions account for more than half of the aromatic-sugar interactions in known structures and are expected to contribute important bond energies, ranging from 1.7 to 6.8 kcal/mol, whereas other interactions (such as OH-π, CH-O, CH-N and almost 30% of unknown interactions) also occur [6].

Within the active site of ODH, both G3G anomers sit with the α-faces of both glucosyl units over the aromatic side chain of Tyr64. Likewise, in ODH-GLC two GLC molecules occupy the equivalent positions of G3G glucosyl units (Figure 6). On the opposite side (sugar β-faces), two well-defined binding sites interact with two pyranose rings separately (when GLC is bound) or simultaneously (when G3G is bound). This mechanism explains the enzymatic preference for G3G (lower K_M) over GLC, deriving from adding up the contributions of both pyranose-protein interactions. More details about the two binding sites follow. Pyranose binding site 1 (reducing end). Above the β-face of the reducing glucosyl unit of both G3G anomers (and above GLC1 in ODH-GLC), ODH active site provides an aromatic platform formed by the two aromatic rings of Trp430 and Phe416, which lie almost on the same plane (Fig. 6). PDBe analysis suggests CH-π plane-plane interactions for the β and α anomers with Trp430 and Phe416 respectively (see Additional file 1, Fig. S9). Trp430 is strictly conserved in proteins belonging to the GOX/GDH clade (see Additional file 1, Fig. S6) and it seems to play the same role in *Af*GDH, as seen in the *Af*GDH-LGC complex (see Additional file 1, Fig. S9), although the authors had excluded CH-π interactions between gluconolactone and this tryptophane [7]. Both α–GLC1 and α–G3G reducing end bind to Phe416 with an almost ideal plane-plane stacking, which is in line with previously reported data. In fact, while β-D-glucopyranose can engage in CH-π interactions with aromatic side chains with both the α and the β pyranose faces (each having three -CH groups available for binding) [8], α-D-glucopyranose favors binding with its β face (providing four -CH groups) rather than the α face (having only two -CH moieties left and the O1 hydroxyl in unfavorable position) [5]. Phe416 might play a role in discriminating between α- and β-D-glucopyranose, due to its preference for the former, in line with the activities measured on different anomers (see Additional file 1, Fig. S5). This residue is conserved in the ODH and ODH-like subclades, and to a certain extent in GOX and GOX-like enzymes (see Additional file 1, Fig. S6). Pyranose binding site 2 (non-reducing end). The β-face of G3G non-reducing glucosyl units (and that of GLC2 in ODH-GLC) is also engaged in CH-π interactions with Phe421 (see Additional file 1, Fig. S9), whose phenyl ring stacks to the pyranose ring of GLC2 and of G3G non-reducing end (Fig. 6, and see Additional file 1, Fig. S8). This residue belongs to the substrate-binding loop, as described above, and probably triggers the large loop displacement upon substrate recognition.

Section 4. The puzzling role of Tyr64 (sugar α-face)

Within the active site of ODH, both G3G anomers, as well GLC1 and 2, sit with their pyranose α-faces over the aromatic side chain of Tyr64. This residue is well conserved within the GOX/GDH clade of AA3_2 enzymes, except for GOX-like proteins, due to a gene deletion of approximately 27 amino acids (see Additional file 1, Fig. S6). In GDH class-II enzymes, other amino acids (Phe and Trp) are accepted, although they bear no hydroxyl on their sidechains. In ODH-GLC, a water molecule bridges the β-GLC1 O3 hydroxyl to Tyr64 hydroxyl (Fig. 6). However, despite the Tyr64 equivalent in *Af*GDH (Tyr53) has been suggested to be involved in substrate stabilization via hydrogen bonds [7], PDBe analysis suggests only van der Waals interactions with Tyr53 hydroxyl in *Af*GDH-LGC and no hydrogen bonds with Tyr64 in ODH-G3G (see Additional file 1, Fig. S9); in this structure instead, Tyr64 hydroxyl establishes a weak hydrogen bond (CH-O) with the C3 of β-G3G (see Additional file 1, Fig. S9), and other weak electrostatic and van der Waals interactions are established with Cε and Cζ of the tyrosine side chain (not shown).

Altogether, these data suggest that Tyr64 may have a role in substrate binding, either through polar or non-polar interactions, but not necessarily through hydrogen bonds between sugar and tyrosine hydroxyls.

References

1. Golovin A, Henrick H. MSDmotif: exploring protein sites and motifs. BMC Bioinformatics. 2008;9:312.
2. Nishio M. The CH/π hydrogen bond in chemistry. Conformation, supramolecules, optical resolution and interactions involving carbohydrates. Phys Chem Chem Phys. 2011;13:13873-900.
3. Nishio M, Umezawa Y, Fantini J, Weissd MS, Chakrabartie P. CH–π hydrogen bonds in biological macromolecules. 2014;16:12648-83.
4. Brandl M, Weiss MS, Jabs A, Sühnel J, Hilgenfeld R. C-H...pi-interactions in proteins. J Mol Biol. 2001;307:357-77.
5. Spiwok V. CH/π Interactions in Carbohydrate Recognition. Molecules. 2017;22:1038.
6. Stanković IM, Blagojević Filipović JP, Zarić SD. Carbohydrate - Protein aromatic ring interactions beyond CH/π interactions: A Protein Data Bank survey and quantum chemical calculations. Int J Biol Macromol. 2020;157:1-9.
7. Yoshida H, Sakai G, Mori K, Kojima K, Kamitori S, Sode K. Structural analysis of fungus-derived FAD glucose dehydrogenase. Sci Rep. 2015;5:13498.
8. Rose IA, Hanson KR, Wilkinson KD, Wimmer MJ. A suggestion for naming faces of ring compounds. PNAS. 1980;77:2439-41.
